# Genetic diversity of macrolides resistant *Staphylococcus aureus* clinical isolates and the potential synergistic effect of vitamins, C and K_3_

**DOI:** 10.1186/s12866-023-03169-1

**Published:** 2024-01-20

**Authors:** Tarek El-Said El-Banna, Fatma Ibrahim Sonbol, Amal M. Abo Kamer, Sara Ahmed Mohammed Mahmoud Badr

**Affiliations:** Faculty of Pharmacy, Tanta University, Tanta, Egypt

**Keywords:** *Staphylococcus aureus*, *Erm genes*, *Macrolides*, *Vitamin C*, *Vitamin K*_3_

## Abstract

**Background:**

Macrolide antibiotics have been extensively used for the treatment of *Staphylococcus aureus* infections. However, the emergence of macrolide-resistant strains of *S. aureus* has become a major concern for public health. The molecular mechanisms underlying macrolide resistance in *S. aureus* are complex and diverse, involving both target site modification and efflux pump systems. In this study, we aim to overcome the molecular diversity of macrolide resistance mechanisms in *S. aureus* by identifying common molecular targets that could be exploited for the development of novel therapeutics.

**Methods:**

About 300 *Staphylococcus aureus* different isolates were recovered and purified from 921 clinical specimen including urine (88), blood (156), sputum (264), nasal swabs (168), pus (181) and bone (39) collected from different departments in Tanta University Hospital. Macrolide resistant isolates were detected and tested for Multi Drug Resistant (MDR). Gel electrophoresis was performed after the D test and PCR reaction for *erm(A), (B), (C), msr(A), and mph(C)* genes. Finally, we tried different combinations of Erythromycin or Azithromycin antibiotics with either vitamin K_3_ or vitamin C.

**Results:**

Macrolide resistance *S. aureus* isolates exhibited 7 major resistance patterns according to number of resistance markers and each pattern included sub patterns or subgroups. The PCR amplified products of different *erm* genes; analysis recorded different phenotypes of the *Staphylococcus aureus* isolates according to their different genotypes. In addition, our new tested combinations of Erythromycin and vitamin C, Erythromycin, and vitamin K_3_, Azithromycin and vitamin C and Azithromycin and vitamin K_3_ showed significant antibacterial effect when using every antibiotic alone. Our findings provide new insights into the molecular mechanisms of macrolide resistance in *S. aureus* and offer potential strategies for the development of novel protocols to overcome this emerging public health threat.

**Supplementary Information:**

The online version contains supplementary material available at 10.1186/s12866-023-03169-1.

## Introduction

*Staphylococcus aureus* is a gram-positive bacterium that is commonly found on the skin and in the nasal passages of healthy individuals. While it is a normal part of the human microbiota, it has the potential to cause a wide range of infections, ranging from minor skin infections to life-threatening conditions, such as sepsis and endocarditis [1, 2]. One of the key factors that contribute to the pathogenicity of *S. aureus* is its ability to produce a range of virulence factors, including toxins, enzymes, biofilm and adhesins, which allow it to colonize and invade host tissues. For example, the bacterium produces a protein called coagulase that allows it to form clumps in the bloodstream, which can lead to the formation of blood clots and the development of abscesses [3–5].

*S. aureus* is also able to evade the immune system by producing proteins that inhibit the function of white blood cells and other immune cells. Additionally, it has the ability to acquire resistance to antibiotics through the acquisition of resistance genes, making it a significant public health concern [6–8].

Although chemically different, macrolide and lincosamide antibiotics have a similar method of action [9]. Their range of activity is restricted to gram-positive cocci and bacilli (mostly staphylococci and streptococci), gram-negative cocci, and intracellular bacteria (species of Chlamydia and Rickettsia) [10]. A few significant exceptions to the general rule that Gram-negative bacilli are resistant include the species of *Bordetella pertussis, Campylobacter, Chlamydia, Helicobacter*, and *Legionella* [11, 12].

Macrolides are made up of two neutral or amino sugars joined to a lactone ring with a range of sizes. The 14-membered (clarithromycin, dirithromycin, erythromycin, and roxithromycin) or 15-membered (azithromycin) lactone ring is present in all commercially marketed macrolides. In some nations or in veterinary medicine (tylosin), sixteen-membered ring macrolides (josamycin, midecamycin, miocamycin, rokitamyin, and spiramycin) are accessible. Clindamycin and lincomycin are lincosamides that lack a lactone ring [13, 14].

It has been documented that staphylococcus started developing macrolide resistance soon after erythromycin was used therapeutically [15, 16]. Most often, target site change caused by methylation of adenosine 2058 (A2058) of the 23S rRNA inside the large ribosomal subunit has been related to macrolide resistance in clinical isolates of Staphylococci [17, 18]. *erm* genes produce these ribosomal methylases. Erythromycin, clarithromycin, roxithromycin, and dirithromycin are examples of 14-member-ring macrolides that are actively effluxed by ABC transporters expressed by plasmid-borne *msr(A)* genes. Azithromycin is an example of a 15-member-ring macrolide [19–21]. According to reports [22, 23], a small number of staphylococcal strains can develop a macrolide phosphotransferase that renders some of these antibiotics inactive. Drug efflux and ribosomal methylation have generally been identified to be the causes of macrolide resistance in all strains investigated in many survey investigations. [24, 25].

Knowledge about the emerge of resistance of macrolides among *S. aureus* is the aim goal of the present study to develop the hypothesis of prevention and control measures of infection caused by this bacterial pathogen.

## Material and methods

### Microorganisms

A total of 300 *Staphylococcus aureus* isolates were recovered from 921 clinical specimens including urine (88), blood (156), sputum (264), nasal swabs (168), pus (181), ICU (25) and Bone (39) collected from different departments of Tanta university hospital.

### Isolation and purification of bacteria

According to [26], Staphylococci isolates were isolated and identified. The blood culture broth was subcultured on nutrient agar plate after being incubated at 37°C for 24 h. The nutrition agar plate was directly streaked with one loopful of the urine sample. The generated bacterial colonies on culture plates were morphologically assessed for their size, shape, elevation, margin, color, consistency, and transparency after an overnight incubation at 37°C. After the Gram staining, the obtained bacterial isolates were inspected under a microscope. Each isolate was put through additional biochemical testing in accordance with its arrangement, shape, and Gram reaction.

#### Biochemical tests for identification of staphylococci

Catalase test, Coagulase test, Deoxyribonuclease (DNase test) and Mannitol fermentation test were carried out according to [26]. The suspected isolates were streaked on LSM agar, according to [27].

#### Antibiotics resistance screening of the isolated Staphylococci and Multi Drug Resistant (MDR) isolates

Disc agar diffusion was used to test the staphylococci clinical isolates for resistance to various antibiotics in accordance with [28]. On Muller Hinton agar plates, bacterial inoculum was cultured and the discs of tested antimicrobial agents of erythromycin, azithromycin, clarithromycin, clindamycin, streptogramin B, penicillin, cefoxitin, rifampin, oxacillin, vancomycin, ciprofloxacin, nitrofurantoin, cotrimoxazole, gentamycin, methicillin, tetracycline, moxifloxacin and chloramphenicol were applied and incubated at 37°C for 24 h.

The MAR index values for each isolate and each antibiotic were calculated, according to [29] using the formulas:

$$\mathrm{MAR index for antibiotics }=\frac{\mathrm{Number of antibiotic resistant isolates}}{\mathrm{Number of antibiotics x number of isolates}}$$

$$\mathrm{MAR index for isolates }=\frac{\mathrm{Number of antibiotics to which the isolate was resistant }}{\mathrm{Total number of antibiotics to which the isolate was exposed}}$$

#### Antibiotics MIC detection

According to the process outlined by [30], MICs of macrolide-resistant staphylococci isolates were assessed by the agar dilution method. The presence of growth on the plate containing the least amount of the specified antimicrobial agent after (18–24) hours of incubation at 37°C reveals the minimum inhibitory concentration (MIC) value of this antimicrobial for the tested isolate.

### Studying the resistance mechanisms of macrolides resistant Staphylococcus aureus isolates

#### Detection of erm gene in macrolides resistant Staphylococcus aureus isolates using the disk approximation test (D-test)

According to the procedure described by [28], the disc approximation test (D test) was used to detect inducible clindamycin resistance caused by the presence of the erm gene. A disc containing 2 g of clindamycin and 15 g of erythromycin were placed 20 mm apart as part of the procedure.

#### Molecular analysis of Macrolides resistant Staphylococcus aureus isolates

##### DNA extraction of *Staphylococcus aureus* isolates

The DNA of Macrolides resistant *Staphylococcus aureus* isolates was extracted using QIAamp® DNA Mini Kit QIAGEN (Germany).

##### PCR for the isolated bacterial DNA

Genes for *ermA, ermB, ermC, msrA*, and *mphC* were amplified using whole DNA extract from various isolates. Each PCR reaction tube held 12.5 µl of the 2X PCR master mix, 2 µl of DNA solution, 1 µl of the forward primer, 1 µl of the reverse primer, and 8.5 µl of nuclease-free water to complete the 25 µl reaction.

The PCR procedures for the *erm* genes were carried out in accordance with the instructions provided by [31] and [32].

##### Agarose gel electrophoresis

Gel electrophoresis for the PCR product and *erm* genes visualization and detection were carried according to [33].

### Investigation of the effect of drugs {Vitamin C (Ascorbic acid) & Vitamin k_3_ (Menadione)} / macrolides combinations on Macrolides resistant Staphylococcus aureus isolates:

#### Determination of MIC of tested drugs / Macrolides combinations on Macrolides resistant Staphylococcus aureus isolates

MIC of tested drugs / macrolides combinations against Macrolides resistant *Staphylococcus aureus* isolates was determined by agar dilution methods, according to the procedure described by [30].

#### Evaluation the effect of the tested drugs combination and different macrolide agents

To examine the impact of combining tested medications with macrolide agents on 80 typical macrolides resistant *Staphylococcus aureus* isolates, tested drugs (Ascorbic acid and vitamin K_3_) were chosen. Each medication was dissolved in a solution with concentrations of (0.5 / 0.25 / 0.125 MIC) and added to the basal medium. Using MH broth in microliter plates, the checkerboard titration method was first used to test each combination [34].

The antimicrobial activity of the tested drugs—Macrolide agents combination was interpreted as one of the following categories: (where the Synergistic effect (Syn A) was detected when Fractional inhibitory concentration index (FICI) value ≤ 0.5; a commutative effect (ADD) when FICI value > 0.5—1; an indifferent effect ((Ind A) at FICI value (1–4) and an antagonistic effect (Ant A) at FICI value > 4 according to FIC equation [34]

$${\text{FIC}}=\frac{\mathrm{MIC of drugs or antibiotic in combination}}{\mathrm{MIC of drug or antibiotic alone}}$$

$${\text{FICI}}=\mathrm{FIC of drug }+\mathrm{ FIC of antibiotics}$$

## Results

### Isolation and identification of staphylococcus aureus

**Fig. 1:**
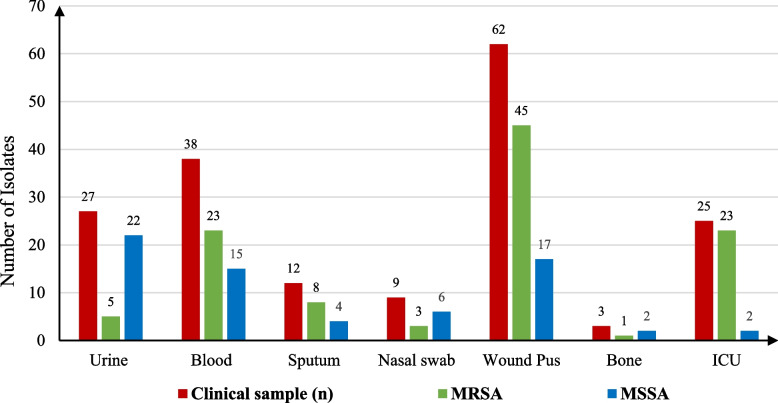
The distribution of the recovered macrolides resistant *Staphylococcus aureus* isolates in different clinical samples and showed the Frequency of MRSA and MSSA according to different clinical samples origin. MRSA: methicillin resistant *Staphylococcus.aureus.* MSSA: methicillin resistant *Staphylococcus.aureus*

A total of 921 clinical samples including urine (88), ICU (25), bone (39), blood (156), sputum (264), nasal swabs (168) and wound pus (181) were obtained from different departments in Tanta University Hospital. All samples were cultured on nutrient agar and examined microscopically. A gram-positive bacteria were subjected to biochemical identification which revealed that 399 isolates were staphylococci. About 300 of these isolates were *S. aureus* and the remaining 99 were CoNS. The distribution of the recovered macrolides resistant *Staphylococcus aureus* isolates in different clinical samples and showed the frequency of MRSA and MSSA according to different clinical samples origin as shown in Fig. 1.

All the *S. aureus* isolates were able to grow on MSA and showed D-mannitol fermentation demonstrated by the production of yellow colonies and haloes. They appeared under the microscope as Gram-positive cocci arranged in grape like structure. Moreover, they were catalase positive with immediate effervescence formation. In addition, all isolates were coagulase positive produced plasma clot that remained in place after inverting the tube. Moreover, all isolates were DNase positive with the formation of clear zone around the spot culture, and also, were protease positive formed clear zone around growth on casein culture media.

### Screening of macrolide resistance and MIC determination of the Staphylococcus aureus isolates

All the recovered (300) staphylococcus aureus isolated were subjected to disk agar diffusion method for macrolide resistant *Staphylococcus aureus* identification. About 176 isolates were macrolide resistant and about 124 isolates were sensitive. MLSB resistant *S. aureus* MIC values for macrolides ranged from less than 4 to 1024 µg/ml and the macrolide breakpoint (≥ 8) ug/ml according to [30] (Table 1).

**Table 1:** MIC values of different macrolides antibiotics

Erythromycin MICS ug/ml	No of resistant isolates	Azithromycin MICS ug/ml	No of resistant isolates	Clarithromycin MICS ug/ml	No of resistant isolate	Clindamycin MICS ug/ml	No of resistant isolate
32	6R	8:16	6R	16	3R	16	23R
64	2R	32	2R	32	4R	32	6R
128	2R	64: 128	-	128	5R	64	7R
256	2R	256	7R	256	3R	128	26R
512	8R	512	13R	512	5R	256	3R
1024	57R	1024	33R	1024	18R	512: 1024	10R
≤ 0.5	25 sensitive	≤ 0.5	11 sensitive	≤ 0.5	13 Sensitive	≤ 0.5	60 Sensitive
1–4	35 intermediate	1–4	13 intermediate	1–4	27 intermediate	1–2	20 intermediate
Total no of resistant strain	77	Total no of resistant strain	61	Total no of resistant strain	38	Total no of resistant strain	75

*MIC* Minimum inhibitory concentration, *R* Resistant isolates

### Susceptibility of macrolide resistant S. aureus isolates to different antimicrobial agents:

**Fig. 2:**
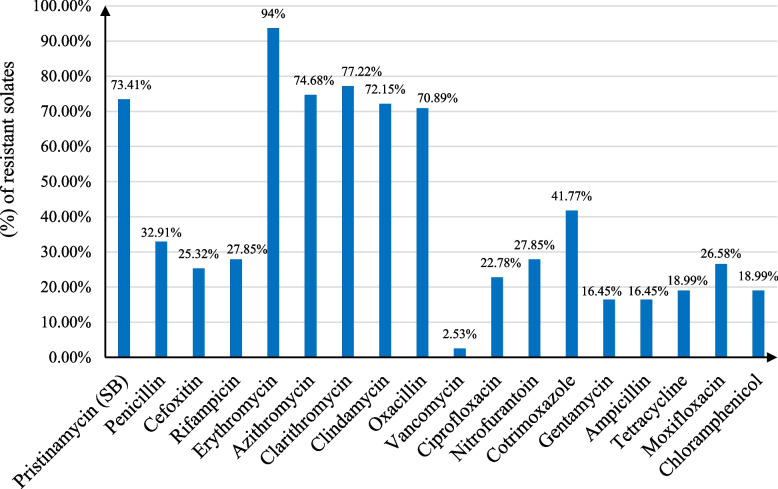
Incidences of resistance to different antimicrobial agents in macrolide resistant *S. aureus* (*n* = 79)

The susceptibility of 79 isolates to 18 different antimicrobial agents were selected according to the highest resistance recorded (≥ 1024) and performed using disk diffusion method except for *vancomycin The concentration used was* 30 µg which is compared with CLSI standards of sensitivity by disk diffusion, then we confirmed the result by making susceptibility testing breakpoints, where's (CLSI) states that MIC ≥ 16 µg/ml should be regarded as resistance.so we use a 16 µg/ml of vancomycin powder to confirm the result that showed two vancomycin resistant isolates (2.53%*) and the* Incidences of resistance to different antimicrobial agents in macrolide resistant *S. aureus* (n = 79) showed in Fig. 2.

### Antimicrobial resistance pattern of macrolide resistant Staphylococcus aureus isolates and MDR detection

Macrolide resistance *S. aureus* isolates exhibited 7 major resistance patterns according to number of resistance markers and each pattern included sub patterns or subgroups. All isolates were resistant to up to 3–15 out of the tested 18 antimicrobial agents. Tested isolates were very heterogeneous where not more than 7 isolates shared the same resistance pattern.

The isolate that showed resistance to at least one agent in ≥ 3 antimicrobial categories was considered MDR, as shown in Table 2.

**Table 2:** Antimicrobial resistance patterns of macrolide resistant *S. aureus* isolates

Markers number	Pattern code	Antimicrobial resistance pattern*	No. (%) of isolates	Resistance profile	MAR Index
3	PI a	RP, OX, Azt	5 (6.33%)	MDR	0.16
	PI b	RP, ery, Cot	5 (6.33%)	MDR	0.16
5	PII a	Cot, Clr, Azt, ery, RP	3 (3.79%)	MDR	0.28
	PII b	Ery, OX, Clr, RP, NIT, Cot	2 (2.53%)	MDR	0.28
	PII c	Ery, azt, Clr, CD, NIT	4 (5.06%)	MDR	0.28
6	PIII a	Ery, azt, CD, CLR, RP, ox	4 (5.06%)	MDR	0.33
	PIII b	Ery, azt, Dr, CD, RP, TE	2 (2.53%)	MDR	0.33
	PIII c	Ery, azt, Clr, CD, RP, Mox	3 (3.79%)	MDR	0.33
	PIII d	Ery, azt, Clr, CD, RP, OX	3 (3.79%)	MDR	0.33
	PIII e	Ery, azt, Clr, CD, OX, RP	5 (6.33%)	MDR	0.33
	PIII f	Ery, azt, Clr, RP, cx, ox	4 (5.06%)	MDR	0.33
7	PIV a	Ery, azt, Clr, CD, OX, RIF, RP	3 (3.79%)	MDR	0.39
	PIV b	Ery, azt, Clr, CD, NIT, RP, OX	3 (3.79%)	MDR	0.39
	PIV c	Ery, azt, Clr, CD, OX, RP, P	4 (5.06%)	MDR	0.39
8	PV a	Ery, azt, Clr, RP, P, Cx, OX, Cot	3 (3.79%)	MDR	0.44
	PV b	Ery, azt, Clr, CD, RIF, RP, ox, P	3 (3.79%)	MDR	0.44
9	PVI a	Ery, azt, Clr, CD, OX, RP, RIF, P, NIT	3 (3.79%)	MDR	0.5
	PVI b	Ery, azt, Clr, CD, RP, VA, COT, OX, C	2 (2.53%)	MDR	0.5
	PVI c	Ery, azt, Clr, CD, RP, OX, CIP, COT, MOX	5 (6.33%)	MDR	0.5
15	PVII a	Amp, P, C X, RIF, Ery, Clr, CD, Ox, Cip, NIT, Cot, Gen, Tet, Mox, C	7 (8.86)	MDR	0.8
	PVII b	Amp, P, CX, RIF, Ery, RP, CD, Ox, Cip, NIT, Cot, Gen, Tet, Mox, c	6 (7.59)	MDR	0.8

*ERY* Erythromycin, *CD* Clindamycin, *Rp* Streptogramin B, *P* Penicillin, *CX* Cefoxitin, *RIF* Rifampicin, *AZT* Azithromycin, *CLR* Clarithromycin, *OX* Oxacillin, *VA* Vancomycin, *CIP* Ciprofloxacin, *NIT* Nitrofurantoin, *COT* Cotrimoxazole (Trimethoprim-sulfamethoxazole), *GEN* Gentamycin, *AMP* Ampicillin, *TE* Tetracycline, *MOX* Moxifloxacin, *C* Chloramphenicol, *(MDR)* Multi Drug Resistant

### Studying the resistance mechanisms of certain antimicrobials among macrolide resistant S. aureus isolates

#### Detection of the erm gene in the macrolides resistant isolated Staphylococcus aureus using disc approximation test (D-test)

*S. aureus* isolates resistant to erythromycin and sensitive to clindamycin were screened.

**Fig. 3:**
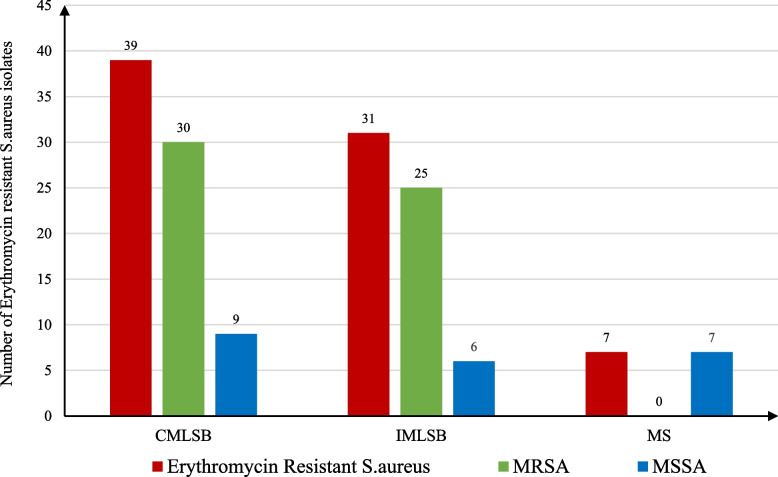
The distribution of erythromycin resistance phenotypes among MRSA & MSSA. MLSB = Macrolides lincosamide streptogramin B. MS or NEG = Macrolide streptogramin B resistant and Negative for clindamycin. MRSA = Methicillin resistant *Staphylococcus aureus*. MSSA = Methicillin sensitive *Staphylococcus aureus*. MS or NEG = Macrolide streptogramin B resistant and Negative for clindamycin. IMLSB = inducible resistance in Macrolides lincosamide streptogramin B. CMLSB = constitutive resistance in Macrolides lincosamide streptogramin B

The growth of the organism up to the edges of the disc, flattening of the clindamycin zone (D test positive) near the erythromycin disc (resistant) and susceptible to both antibiotics implicate that the organism is having constitutive MLSB (CMLSB), inducible MLSB (IMLSB) and no resistance respectively. Further, the organism susceptible to clindamycin without any flattening of the zone (D test negative) near clindamycin disc (resistant) implicates that the organism is having macrolide streptogramin resistance (MSB). The gene encodes enzymes that confer inducible resistance to lincosamides (clindamycin) via methylation of the 23S rRNA were recorded and the distribution of erythromycin resistance phenotypes among MRSA & MSSA is shown in Fig. 3.

### Erythromycin and Azithromycin combination with Vitamins C and K_3_ effect

#### Erythromycin and Vitamin K3 combination effect

When the MICs of erythromycin and vitamin K together were at least four times lower than MICs of erythromycin alone, there was a stronger antibacterial impact. Against various Staphylococcus aureus isolates, the menadione showed a MIC = (64 & 128) ug/mL.

Erythromycin synergistic effect with VIT K_3_ resulted in a reduction of the MICs by ≥ fourfold in most of *S. aureus* tested, evidencing a synergistic effect as defined by a FICI of ≤ 0.5, for example, with some isolates, such as 80, 317, and 102 had synergistic effect, that finally demonstrated a strong antimicrobial activity of vit. K_3_ as shown in Table 3.

**Table 3:** The antimicrobial effect of Erythromycin combination with vit K_3_

Sample code	Mic of Vit K Alone	Mic of ERY Alone	$\frac{1}{2}$ mic of Vit K + ERY	FICI	$\frac{1}{4}$ mic of Vit K + ERY	FICI	$\frac{1}{8}$ mic of Vit K + ERY	FICI
27 A	64	1024	0.5	0.5 S	0.5	0.25 S	0.5	0.12 S
80	64	1024	0.5	0.5 S	0.5	0.25 S	0.5	0.12 S
18^+^	64	1024	0.5	0.5 S	0.5	0.25 S	0.5	0.12 S
317	64	1024	0.5	0.5 S	0.5	0.25 S	0.5	0.12 S
102	64	32	0.5	0.51 S	0.5	0.26 S	0.5	0.14 S
305	64	1024	128	0.62 ADD	256	0.5 S	512	0.62 ADD
316	64	1024	128	0.62 ADD	512	0.75 ADD	1024	1.12 IND
7^+^	64	1024	0.5	0.5 S	0.5	0.25 S	0.5	0.12 S
83	64	32	0.5	0.51 S	0.5	0.26 S	0.5	0.14 S
58	64	1024	0.25	0.5 S	0.5	0.25 S	0.5	0.12 S
44 A	64	1024	0.25	0.5 S	0.5	0.25 S	0.5	0.12 S
G	64	1024	0.5	0.5 S	0.5	0.25 S	0.5	0.12 S
307	64	1024	1024	1.5 IND	1024	1.25 IND	1024	1.12 No
324	64	1024	512	1 Ad	1024	1.25 No	1024	1.12 No
316	64	1024	1024	1.5 IND	1024	1.25 IND	1024	1.12 No
75 A	64	1024	0.5	0.5 S	0.5	0.25 S	0.5	0.12 S
310	64	1024	8	0.5 S	512	0.75 Ad	1024	1.12 IND
308	64	512	256	1 AD	512	1.25 IND	1024	2.12 IND
41 A	64	1024	0.5	0.5 S	0.5	0.25 S	0.5	0.12 S
53	64	32	0.5	0.5 S	0.5	0.26 S	0.5	0.14 S
303	64	1024	512	1 Ad	1024	1.25 IND	1024	1.12 IND
69 A	64	512	0.5	0.5 S	0.5	0.25 S	0.5	0.12 S
78	64	1024	128	0.62 AD	256	0.5 S	512	0.62 AD
321	64	1024	128	0.62 AD	512	0.75 AD	1024	1.12 IND
Z	64	1024	1024	1.5 IND	1024	1.25 IND	1024	1.25 IND
300^+^	64	512	1024	2.5 IND	1024	2.25 IND	1024	2.125 IND
38 A	64	1024	1024	1.5 IND	1024	1.25 IND	1024	1.25 IND
100 A	64	1024	2	0.5 S	8	0.25 S	16	0.14 S
39 A	128	1024	2	0.5 S	2	0.25 S	2	0.12 S
C	128	1024	1024	1.5 IND	1024	1.25 IND	1024	1.125 IND
28	128	32	2	0.56 AD	4	0.37 S	16	0.625 AD
90^+^	128	16	0.5	0.53 S	0.5	0.28 S	32	2.125 IND
40 A	128	256	0.5	0.5 S	0.5	0.251 S	1024	4.125 ANTA
204	128	512	0.5	0.5 S	0.5	0.25 S	0.5	0.12 S
B1	128	128	0.25	0.5 S	0.25	0.25 S	0.5	0.12 S
32 A	128	1024	0.25	0.5 S	0.5	0.25 S	0.5	0.125 S
45 A	128	1024	0.25	0.5 S	0.25	0.25 S	0.5	0.125 S
43 A	128	1024	0.25	0.5 S	0.25	0.25 S	0.5	0.125 S
100	128	1024	0.25	0.5 S	0.25	0.25 S	1	0.125 S
46 A	128	1024	4	0.5 S	8	0.25 S	16	0.14 S
67 A	128	1024	2	0.5 S	16	0.26 S	128	0.25 S
M	128	1024	2	0.5 S	16	0.26 S	128	0.25 S
202	128	1024	1024	1.5 IND	1024	1.25 IND	1024	1.25 IND
22	128	1024	512	1 IND	512	0.75 AD	1024	1.125 IND
90	128	1024	2	0.5 S	2	0.25 S	2	0.126 S
89 +	128	1024	16	0.5 S	32	0.28 S	512	0.62 Add
C	128	1024	512	1 AD	1024	1.25 IND	1024	1.25 IND
F	128	1024	64	0.5 S	1024	1.25 IND	1024	1.25 IND
89	128	1024	2	0.5 S	512	0.75 AD	1024	1.125 IND
71 A	128	1024	1024	1.25 IND	1024	1.25 IND	1024	1.125 IND
T	128	512	16	0.5 S	256	0.75 Ad	512	1.125 IND
A	128	1024	16	0.5 S	512	0.75 AD	512	0.62 AD
300	128	512	32	0.5 S	64	0.375 S	128	0.375 S
28 A	128	1024	0.5	0.5 S	0.5	0.25 S	0.5	0.125 S
93^+^	128	1024	2	0.5 S	16	0.26 S	32	0.15 S

*S* Synergism, *ADD* ADDITIVE, *NO* NO difference and *ANTA* Antagonism, *FICI* Fractional inhibitory concentration index, *MIC* Minimum inhibitory concentration index, *Ery* Erythromycin

#### Combination effect of Azithromycin (Azt) and Vitamin K_3_

When the MICs of azithromycin in conjunction with vitamin K were at least four times lower than the MICs of azithromycin alone, the antibacterial impact was increased. Against various *Staphylococcus aureus* isolates, the menadione showed a MIC = 64 ug/mL. The interaction effect of Azithromycin in combination with VIT. K resulted in a reduction of the MICs by ≥ fourfold in most of *S. aureus* tested, evidencing a synergistic effect as defined by a FICI of ≤ 0.5, for example, with some isolates, such as 80, 58, and 324 had synergistic effect, which finally demonstrated strong antimicrobial activity of vit. K_3_ as shown in Table 4.

**Table 4:** Antimicrobial effect of combination between the vit K_3_ and Azythromycin

Sample code	Mic of Vit.K Alone	Mic of AZt Alone	$\frac{1}{2}$ mic of Vit.K + AZt	FICI	$\frac{1}{4}$ mic of Vit.K + AZt	FICI	$\frac{1}{8}$ mic of Vit.K + AZt	FICI
27 A	64	1024	256	0.75 ADD	512	0.75 ADD	1024	1.12 IND
80	64	1024	2	0.5 S	4	0.25 S	8	0.13 S
18^+^	64	1024	1	0.5 S	2	0.25 S	4	0.13 S
317	64	512	2	0.5 S	512	1.25 IND	1024	2.125 IND
102	64	512	2	0.5 S	8	0.26 S	16	0.15 S
305	64	512	2	0.5 S	8	0.26 S	16	0.15 S
316	64	1024	1024	1.5 IND	1024	1.5 IND	1024	1.12 IND
7^+^	64	1024	1	0.5 S	2	0.25 S	8	0.132 S
83	64	16	0.5	0.5 S	0.5	0.28 S	32	2.1 IND
58	64	1024	1	0.5 S	2	0.25 S	4	0.13 S
44 A	64	1024	1	0.5 S	2	0.25 S	8	0.132 S
G	64	1024	0.5	0.5 S	1	0.25 S	2	0.126 S
307	64	1024	1024	1.5 IND	1024	1.5 IND	1024	1.12 IND
324	64	1024	0.5	0.5 S	1	0.25 S	2	0.12 S
316	64	512	1024	2.5 IND	2048	4.25 ANT	2048	4.12 ANT
75 A	64	1024	1024	1.5 IND	1024	1.25 IND	1024	1.12 IND
310	64	1024	8	0.5 S	4	0.25 S	2	1.126 IND
308	64	16	0.5	0.53 S	1	0.31 S	2	0.25 S
41 A	64	512	1024	2.5 IND	1024	2.25 IND	1024	2.1 IND
53	64	1024	0.5	0.5 S	1	0.25 S	2	0.12 S
303	64	1024	16	0.5 S	32	0.28 S	64	0.18 S
69 A	64	512	512	1.5 IND	1024	2.25 IND	1024	2.12 IND
78	64	1024	1024	1.5 IND	1024	1.25 IND	1024	1.125 IND
321	64	512	2	0.5 S	512	1.25 IND	1024	2.125 IND
46A	64	1024	1024	1.5 IND	1024	1.25 IND	2	0.12 S
300^+^	64	1024	2	0.5 S	4	0.25 S	8	0.13 S
38 A	64	512	2	0.5 S	8	0.26 S	16	0.15 S
100 A	64	1024	1024	1.5 IND	1024	1.25 IND	1024	1.125 IND
39 A	128	512	2	0.5 S	8	0.26 S	16	0.15 S
T	128	32	0.5	0.5 S	32	1.25 IND	32	1.125 IND
28	128	1024	0.5	0.5 S	1	0.25 S	2048	2.125 IND
90^+^	128	512	0.5	0.5 S	1	0.25 S	2	0.12 S
40 A	128	512	256	1 ADD	512	1.25 IND	512	1.125 IND
204	128	1024	128	0.6 AD	512	0.75 AD	1024	1.125 IND
B1	128	1024	256	0.75 Add	256	0.5 S	256	0.4 S
32 A	128	1024	256	0.75 AD	256	0.5 S	256	0.4 S
45 A	128	8	2	0.75 AD	4	0.75 AD	2	0.37 S
43 A	128	1024	128	0.62 AD	512	0.75 AD	1024	1.1 IND
100	128	512	2	0.5 S	16	0.28 S	32	0.18 S
46 A	128	1024	32	0.5 S	512	0.75 AD	256	0.4 S
M	128	1024	32	0.5 S	512	0.75 ADD	256	0.4 S
4A	128	1024	2	0.5 S	2	0.25 S	4	0.12 S
F	128	1024	32	0.5 S	512	0.75 ADD	256	0.4 S
67 A	128	1024	1024	1.5 IND	1024	1.25 IND	4	0.12 S
202	128	1024	512	1 AD	1024	1.25 IND	1024	1.125 IND
90	128	1024	1024	1.5 IND	1024	1.25 IND	1024	1.125 IND
89 +	128	1024	1024	1.5 IND	2048	2.25 IND	2048	2.125 IND
Z	128	1024	512	1 Ad	1024	1.25 IND	1024	1.125 IND
11A	128	1024	2	0.5 S	2	0.25 S	4	0.12 S
89	128	1024	16	0.51 S	1024	1.25 IND	1024	1.125 IND
71 A	128	256	8	0.53 S	512	2.25 IND	1024	4.12 ANT
93 +	128	1024	512	1 AD	512	0.75 AD	4	0.12 S
A	128	512	32	0.56 ADD	64	0.37 S	512	1.125 IND
300	128	512	0.5	0.5 S	1	0.25 S	2	0.128 S
28 A	128	16	2	0.625 AD	16	1.25 IND	0.5	0.15 S

*S* Synergism, *ADD* ADDITIVE, *NO* NO difference and *ANTA* Antagonism, *FICI* Fractional inhibitory concentration index, *MIC* Minimum inhibitory concentration index, *Azt* Azithromycin

#### Erythromycin and Vitamin C Combination effect

When the MICs of Eryth combined with vitamin C were at least four times lower than the MICs of Ery alone, there was an improved antibacterial action. For example, with some isolates, such as 300, 100, and 58, which had Synergistic effect, the interaction effect of erythromycin in combination with vitamin C resulted in a reduction of the MICs by fourfold in most of the S. aureus tested, evidencing a synergistic effect, demonstrating strong antimicrobial activity of vitamin C as shown in Table 5.

**Table 5:** The antimicrobial effect of Erythromycin combination with vit C

Sample code	Mic of Vit c Alone	Mic Ery Alone	$\frac{1}{2}$ mic of Vit C + Ery	FICI	$\frac{1}{4}$ mic of Vit C + Ery	FICI	$\frac{1}{8}$ mic of Vit C + Ery	FICI
69 A	2000	512	512	1.5 No	1024	2.25 NO	1024	2.125 NO
36	2000	512	128	0.75 AD	256	0.75 AD	256	0.62 AD
103	1000	1024	2	0.5 S	4	0.25 S	8	0.13 S
24	1000	1024	512	1 Add	256	0.5 S	512	0.6 AD
41 A	1000	1024	8	0.5 S	16	0.26 S	32	0.16 S
102	1000	32	2	0.56 AD	2	0.31 S	4	0.25 S
303	1000	1024	1024	1.5 Add	256	0.5 S	1024	1.125 No
39 A	4000	1024	256	0.75 AD	64	0.31 S	128	0.25 S
311	4000	1024	256	0.75 AD	1024	1.2 No	1024	1.12 No
G	2000	1024	32	0.5 S	128	0.38 S	256	0.37 S
307	2000	1024	1024	1.5 No	2048	2.25 NO	2048	2.125 NO
80	2000	1024	1024	1.5 No	2048	2.25 NO	2048	2.125 NO
78 +	2000	1024	1024	1.5 No	2048	2.25 NO	2048	2.125 NO
50 A	2000	1024	1024	1.5 No	2048	2.25 NO	2048	2.125 NO
307	2000	1024	1024	1.5 No	2048	2.25 NO	2048	2.125 NO
302	2000	128	16	0.63 AD	64	0.75 AD	128	1.12 No
82 A	2000	1024	64	0.56 AD	128	0.625 AD	256	0.37 S
40 A	1000	256	8	0.53 SYN	256	1.25 No	512	2.1 NO
44 A	1000	1024	4	0.5 S	8	0.257 S	32	0.156 S
10 A	2000	128	0.5	0.5 S	0.5	0.253 S	0.5	0.3 S
32 A	2000	1024	0.5	0.5 Syn	0.5	0.250 Syn	256	0.375 Syn
67 A	2000	1024	0.5	0.5 Syn	1	0.25 Syn	2	0.13syn
ن	2000	1024	0.5	0.5 S	0.5	0.250 S	0.5	0.125 S
202	2000	1024	256	0.75 AD	256	0.5 S	1024	1.125 No
89 +	2000	1024	256	0.75 AD	1024	1.25 No	1024	1.125 No
86 A	2000	512	0.5	0.5 S	8	0.26 S	64	0.25 S
F	2000	1024	0.5	0.5 S	0.5	0.250 S	0.5	0.125 S
A	2000	1024	0.5	0.5 S	256	0.5 S	1024	1.125 NO
300	2000	512	0.5	0.5 S	0.5	0.251 S	0.5	0.126 S
45 A	2000	1024	0.5	0.5 S	0.5	0.250 S	0.5	0.125 S
28 A	2000	1024	0.5	0.5 S	4	0.25 S	4	0.13 S
300 +	2000	512	0.5	0.5 S	0.5	0.25 S	0.5	0.126 S
43 A	2000	1024	0.5	0.5 S	0.5	0.250 S	0.5	0.125 S
100	2000	1024	0.5	0.5 S	0.5	0.25 S	0.5	0.125 S
100 A	2000	1024	0.5	0.5 S	8	0.275 S	4	0.128 S
48 A	2000	32	0.5	0.51 Syn	4	0.375 S	16	0.6 Add
46 A	2000	1024	0.5	0.5 S	0.5	0.250 S	0.5	0.125 S
4 A	2000	1024	512	1 No	512	0.75 ADD	1024	1.128 No
3 A	2000	1024	0.5	0.5 S	8	0.262 S	512	0.6 ADD
204	2000	1024	0.5	0.5 S	0.5	0.250 S	1	0.12 Syn
58	2000	1024	0.5	0.5 S	0.5	0.250 S	2	0.16 S
56 A	2000	32	0.5	0.51 SYN	4	0.375 S	16	0.6 AD
27 A	2000	1024	0.5	0.5 S	0.5	0.250 S	0.5	0.125 S
89	2000	1024	8	0.50 S	8	0.27 S	8	0.13 S
22	4000	1024	1024	1.5 No	1024	1.25 No	1024	1.12 NO
90	4000	1024	64	0.56 AD	256	0.5 S	512	0.6 AD
38 A	4000	1024	1024	1.5 NO	1024	1.25 No	1024	1.125 NO
78 A	500	1024	1024	1.5 No	2048	2.25 NO	2048	2.125 NO
1 A	500	1024	16	0.5 S	256	0.5 S	2048	2.125 NO
53	500	32	2	0.5 S	2	0.31 S	4	0.25 S

#### Azithromycin and Vitamin C Combination effect

When vitamin C and Azithromycin together had MICs that were at least four times lower than those of Azithromycin alone, there was thought to be an improved antibacterial action. With some isolates, such as 300, 25A, and 311, which had synergistic effect, the interaction effect of azithromycin in combination with vitamin C resulted in a reduction of the MICs by fourfold in most of the S. aureus tested, evidencing a synergistic effect as defined by a FICI of 0.5, demonstrating strong antimicrobial activity of vitamin C as shown in Table 6.

**Table 6:** Antimicrobial effect of combination between the vit C and Azythromycin

Sample code	Mic of Vit c Alone	Mic of AZt Alone	$\frac{1}{2}$ mic of Vit C + AZt	FICI	$\frac{1}{4}$ mic of Vit C + AZt	FICI	$\frac{1}{8}$ mic of Vit C + AZt	FICI
69 A	2000	1024	1024	1.5 No	1024	1.25 No	1024	1.125 No
36	2000	256	64	0.75 AD	256	1.25 No	256	1.25 No
103	1000	512	16	0.53 AD	32	0.31 S	64	0.25 S
24	1000	1024	1024	1.5 No	2048	2.25 NO	2048	2.125 NO
41 A	1000	1024	64	0.5 S	128	0.37 S	256	0.37 S
102	1000	16	8	1 add	16	1.25 No	32	2.125 NO
303	1000	512	1024	2.5 NO	1024	2.25 NO	2048	4.125 Anta
39 A	4000	512	512	1.5 No	1024	2.25 NO	1024	2.125 NO
311	4000	1024	32	0.5 S	64	0.31 S	128	0.25 S
G	2000	512	1024	2.5 NO	2048	4.25 Anta	2048	4.125 Amta
307	2000	1024	1024	1.5 No	2048	2.25 NO	2048	2.125 NO
80	2000	1024	1024	1.5 No	2048	2.25 NO	2048	2.125 NO
78 +	2000	256	1024	4.5 antag	2048	8.25 Antag	2048	8.125 Antag
50 A	2000	512	1024	2.5 NO	2048	4.25 Antag	2048	4.125 Antag
307	2000	512	64	0.6 add	64	0.375 Syn	512	1.125 No
302	2000	1024	32	0.53 SYN	1024	1.25 No	1024	1.125 No
82 A	2000	1024	1	0.5 S	2	0.251 S	4	0.128 S
40 A	1000	8	0.5	0.56 AD	0.5	0.312 S	2	0.315 S
44 A	1000	1024	8	0.50 S	0.5	0.25 S	512	0.5 S
10 A	2000	1024	0.5	0.5 S	0.5	0.5 S	1	0.25 S
32 A	2000	1024	0.5	0.5 S	0.5	0.25 S	1	0.126 S
67 A	2000	1024	256	0.75 AD	256	0.5 S	512	0.625 AD
ن	2000	1024	1024	1.5 No	1024	1.25 No	1024	1.125 No
202	2000	512	256	1 AD	256	0.75 AD	512	0.625 AD
89 +	2000	1024	0.5	0.5 S	1	0.25 S	2	0.126 S
86 A	2000	1024	8	0.50 S	256	0.5 S	512	0.63 ADD
F	2000	512	0.5	0.5 S	0.5	0.250 S	1	0.126 S
A	2000	1024	0.5	0.50 S	0.5	0.25 S	1	0.126 S
300	2000	1024	4	0.5 S	8	0.25 S	1	0.12 S
45 A	2000	512	4	0.5 S	8	0.26 S	1	0.12 S
28 A	2000	1024	8	0.5 S	0.5	0.5 S	1	0.125 S
300 +	2000	512	0.5	0.5 S	1	0.250 S	1	0.126 S
43 A	2000	4	8	0.5 S	8	0.25 S	1024	0.13 S
100	2000	8	16	1 add	8	1.25 No	16	0.62 AD
100 A	2000	8	0.25	0.5 S	2	0.25 S	1024	0.12 S
48 A	2000	256	512	0.75 AD	512	0.75 AD	1024	0.62 AD
46 A	2000	8	256	0.5 S	512	0.75 AD	1024	0.62 AD
4 A	2000	2	4	0.13 S	1	0.26 S	512	0.5 S
3 A	2000	0.5	0.5	0.5 S	1	0.25 S	1024	0.12 S
204	2000	8	16	1 AD	8	1.25 NO	16	0.62 AD
58	2000	8	0.5	0.5 S	2	S 0.25	1024	0.12 S
56 A	2000	4	8	S 0.5	16	0.25 S	1024	0.14 S
27 A	2000	1024	1024	1.5 no	1024	1.25 No	1024	1.125 No
89	2000	1024	1024	1.5 no	1024	1.25 No	1024	1.125 No
22	4000	1024	1024	1.5 No	2048	2.25 NO	2048	2.125 NO
90	4000	1024	1024	1.5 NO	2048	2.25 NO	2048	2.125 NO
38 A	4000	16	2	0.62 AD	2	0.37 S	4	0.37 S
78 A	500	1024	1024	1.5 no	2048	2.25antag	2048	2.125antag
1 A	500	1024	1024	1.5 Add	2048	2.25antag	2048	2.125antag
53	500	16	2	0.62 ADD	2	0.37 S	4	0.37 S

#### Combination effect of Azithromycin and Erythromycin with Vitamin C & K_3_

**Fig. 4:**
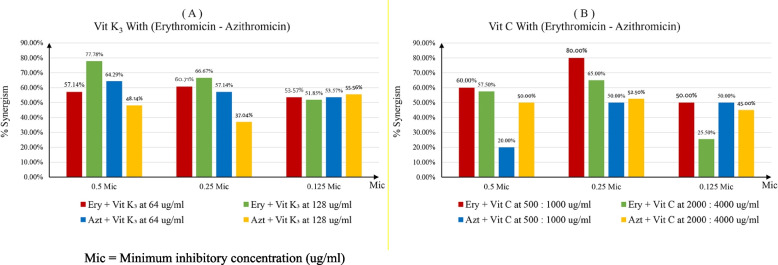
Combination effect of Azithromycin and Erythromycin with Vitamin C & K_3_:. Mic = Minimum inhibitory concentration (ug/ml)

An enhanced antimicrobial effect was recorded after vit k_3_ combination with erythromycin and azithromycin as shown in Fig. 4A. Also, an enhanced antimicrobial effect was recorded after vitamin c combination with erythromycin and azithromycin as shown in Fig. 4B.

### Molecular study of Staphylococcus aureus resistance genes

#### PCR product analysis

For the PCR experiments, representative *Staphylococcus aureus* isolates from Macrolides MICs ranging from 64 to 1024 ug/ml were used. Each chosen isolate's whole DNA extract underwent traditional PCR for the identification of the *erm(A), erm(B), erm(C), msr(A)*, and *mph(C)* genes.

About 25 isolates were tested for the presence of MLSB resistant genes. *erm(A)* was detected in 10 isolates MRSA, and 9 isolates MSSA, while *erm(B)* was detected in 11 MRSA in addition to 8 isolates MSSA. *erm(C)* was detected in 15 MRSA isolates and in 10 of MSSA isolates. Both *erm(A) and erm(B)* were in 10 MRSA and 9 MSSA. Also, both *erm(B) and erm(C)* were detected in 11 MRSA and 8 MSSA. All three genes e*rm(A), erm(B) and erm(C)* were detected in 10 MRSA and 9 MSSA.

The *erm(A)* gene was detected in 19 isolates (76%) (10 isolates MRSA and 9 isolates MSSA), the *erm(C)* gene was detected in 25 isolates (100%) (15 isolates MRSA and 10 isolates MSSA) which was statistically significant (*p* < 0.001) and the *erm(B)* was detected in 19 isolates (76%) (11 isolates MRSA and 8 isolates MSSA). Combination of erm genes was detected in 19 isolates (76%) (10 MRSA and 9 MSSA). All *S. aureus* isolates with MS resistance phenotype (4 isolates) were MSSA and carried the 3 genes (*erm(A), erm(B)* and *erm(B)*). Moreover, most of the isolates with iMLSB resistance phenotype carried both *erm(A)* and *erm(B)* genes (12 isolates, 8 MRSA and 4 MSSA), 8 isolates carried *erm(B)* and *erm(C)* genes and 4 isolates carry *erm(B)* gene only. We found that most of the isolates with cMLSB resistance phenotype were carrying both *erm(B)* and *erm(C)* (13 isolates, 9 MRSA and 4 MSSA) and 12 isolates were carrying *erm(B)* gene. (Table 7).

**Table 7:** Prevalence of *erm* genes in erythromycin resistant strains

Genotype	MRSA	MSSA
*erm(A)*	10 (52.6%)	9 (47.63%)
*erm(B)*	11 (57.89%)	8 (42.1%)
*erm(C)*	15 (60%)	10 (40%)
*erm(A)* + *erm(B)*	10 (52.6%)	9 (47.36%)
*erm(B*) + *erm(C)*	11 (57.89%)	8 (42.1%)
*erm(A)* + *erm(B)* + *erm(C)*	10 (52.6%)	9 (47.63%)

#### PCR for erm gene analysis

##### Molecular detection of erm genes by PCR

**Fig. 5:**
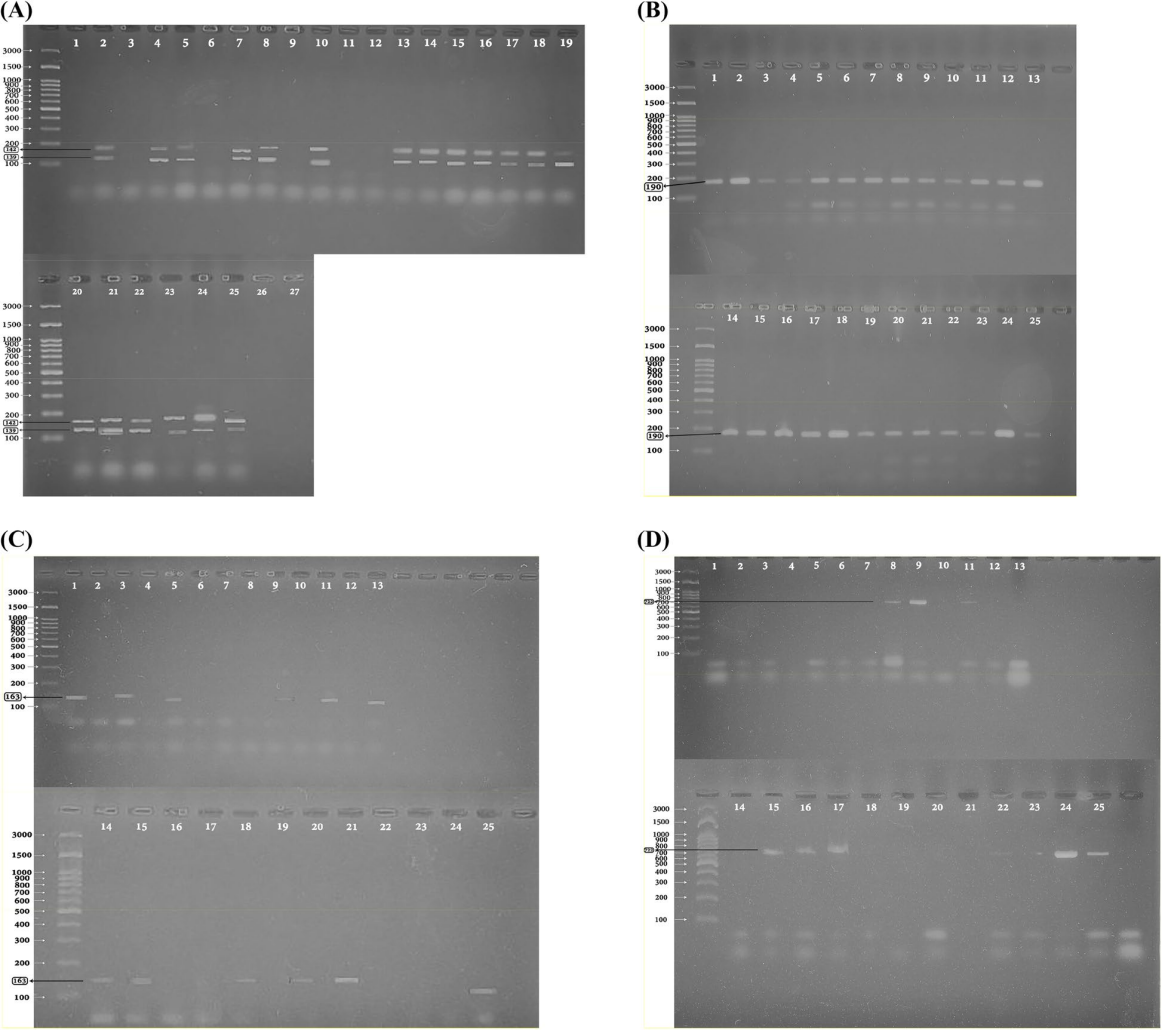
**A** Agarose Gel electrophoresis of (*erm A* & *erm B*) genes at (139, 142bp). ladder lane (m) is 1 Kb, bacterial samples lanes from (1 to 19) are coding for isolates A, 1A, 19, 300, 308, 89^+^, 80, 36, 325, 305, 301, 44A, 3A, & 12A, lanes from (20 to 25) are coding for isolates 28A, 317, 82A, 24, 4A, & 41A respectively. **B** Agarose Gel electrophoresis of *erm(c)* gene at (190bp). ladder lane (m) is 1 Kb. Bacterial samples lanes from (1 to 13) are coding for isolates A, 1A, 19, 300, 308, 89^+^, 80, 36, 325, 305, 301, 44A & 32A, lanes from (14 to 25) are coding for isolates 38A, 45A, 46A, 43A, 3A, 12A, 28A, 317, 82A, 24, 4A & 41A respectively. **C** Agarose gel electrophoresis of *msr(A*) gene at (163bp). Ladder lane (M) is (1 Kb). Bacterial samples lanes from (1 to 13) are coding for isolates A, 1A, 19, 300, 308, 89^+^, 80, 36, 325, 305, 301, 44A & 32A, lanes from (14 to 25) are coding for isolates 38A, 45A, 46A, 43A, 3A, 12A, 28A, 317, 82A, 24, 4A & 41A respectively. **D** Agarose gel of electrophoresis of *mph(c)* gene at (722bp). ladder lane (M) is (1 Kb). Bacterial Samples lanes from (1 to 13) are coding for isolates A, 1A, 19, 300, 308, 89^+^, 80, 36, 325, 305, 301, 44A & 32A, lanes from (14 to 25) are coding for isolates 38A, 45A, 46A, 43A, 3A, 12A, 28A, 317, 82A, 24, 4A & 41A respectively

For the PCR experiments, representative staphylococcus aureus isolates from Macrolides MICs ranging from 64 to 1024 ug/ml were used. Each chosen isolate's whole DNA extract underwent traditional PCR for the identification of the *erm(A), erm(B), erm(C), msr(A)*, and *mph(C)* genes. The amplified products were electrophoresed, and the resulting gels were stained with ethidium bromide and illuminated with ultraviolet light to allow for the visualization of the amplicons. As seen in the electropherogram of both the *erm(A)* and *erm(B)* genes (1–19 & 20–25), bands with approximate sizes of 139 bp for the *erm(A)* gene, 142 bp for the *erm(B)* gene, 190 bp for the *erm(C)* gene, 163 bp for the *msr(A)* gene, and 755 bp for the *mph(C)* gene were founded in (Fig. 5A, B, C, D)**.**

The only three examined isolates in which the five genes were found were isolates S13, 15, and 25. Only ten isolates (S2, 4, 7, 8, 10, 16, 17, 22, 23 and 24) tested positive for *erm(A), (B), and (C).* Additionally, isolates S8, S16, S17, S22, S23, and S24 tested positive for the *mph(C)* gene. Only S9 and S12 isolates showed signs of *erm(C), msr(A)*, and *mph(C)*. Two isolates, S1 and S3, tested positive for *msr(A)* and *erm(C)*. For the gene *erm(C)*, isolates S6 and S12 were positive. Three isolates tested positive for *S14, S20*, and *S21* as well as *erm (A), (B), and(C).* (Table 8).

**Table 8:** PCR result of five genes among 25 Macrolides Resistant *S. aureus*

Total isolates (25)	*erm(A)*	*erm(B)*	*erm(C)*	*msr(A)*	*mph(C)*
S1			+	+	
S2	+	+	+		
S3			+	+	
S4	+	+	+		
S5	+	+	+	+	
S6			+		
S7	+	+	+		
S8	+	+	+		+
S9			+	+	+
S10	+	+	+		
S11			+	+	+
S12			+		
S13	+	+	+	+	+
S14	+	+	+	+	
S15	+	+	+	+	+
S16	+	+	+		+
S17	+	+	+		+
S18	+	+	+	+	
S19	+	+	+		
S20	+	+	+	+	
S21	+	+	+	+	
S22	+	+	+		+
S23	+	+	+		+
S24	+	+	+		+
S25	+	+	+	+	+

It’s concluded from the (Table 8) results that, the highest percentage of the five genes was for *erm(C)* which was detected in all isolates (100%), followed by *erm(A)* and *erm(B)* as each one exhibited (76%) and was detected in nineteen isolates, then *msr(A)* with (48%), and the least gene was *mph(C)* with (44%), as shown in (Table 8).

## Discussion

According to Ullah et al. [35] *Staphylococcus aureus* is one of the most prevalent bacteria that causes both hospital- and community-acquired illnesses. The issue is indicated by resistance to several antimicrobial drugs, which also restricts the available treatments. In the past few years, creating new, efficient medications to treat these infections has been viewed as a crucial new issue in healthcare settings to counteract the shifting patterns of resistance [36].

Clinical isolates of gram-positive bacteria are increasingly being shown to have resistance to macrolides and lincosamides. There are many different phenotypes of resistance due to the diversity of resistance mechanisms, which include ribosomal alteration, antibiotic efflux, and drug inactivation. Clinical relevance of in vitro macrolide resistance is a topic of debate [17, 37].

Due to its availability for parenteral and oral administration, low cost, excellent tissue penetration, and accumulation in abscesses, clindamycin is seen as an intriguing alternative for treating staphylococcal infections [38]*.* According to reports, the formation of MLSB antibiotic resistance bacteria as a result of the abuse of these antibiotics poses a new difficulty for treating these illnesses [39, 40].

In our study, we looked at the mechanisms of *Staphylococcus aureus* macrolides resistance with a focus on the Erm resistance genes. A total of (300) *Staphylococcus aureus* isolates were examined for macrolide-resistant strains. The isolates percentage were (9.5%), (16.9%), (28.66%), (18.24%), (19.65%), (2.82%), (4.23%) from urine, blood, sputum, nasal swaps, pus, ICU and bone, respectively. The frequency of MRSA and MSSA according to different clinical samples origin were (41.67%), (25%), (21.3%), (22%), (4.6%), (32.35%), (7.4%), (5.9%), (2.8%), (8%), (21.3%), (2.9%), (0.9%), (2.9%) from pus, blood, urine, sputum, throat (tracheal samples), ICU and bone respectively. About 124 isolates are sensitive, while about 176 isolates are resistant to macrolides. The total number of isolates were classified according to the MIC values of Erythromycin, Azithromycin, Clarithromycin and Clindamycin to 25 sensitive, 35 intermediate and 77 resistant, 11 sensitive, 13 intermediate and 61 resistant, 13 sensitive, 27 intermediate and 38 resistant and finally, 60 sensitive, 20 intermediate and 75 resistant, respectively.

Macrolide resistance according to the amount of resistance markers*, S. aureus* isolates had 7 major resistance patterns, and each pattern contained sub patterns or subgroups. All isolates were deemed MDR isolated because they were resistant to up to 3–15 of the total of 18 tested antimicrobial drugs. The tested isolates were extremely diverse; not more than five of them which shared the same resistance pattern*. S. aureus* isolates that were susceptible to clindamycin but resistant to erythromycin were chosen for the D test, where they were divided into 3 distinct phenotypes based on the results.

*erm(A)* was detected in 10 isolates MRSA, and 9 isolates MSSA, while *erm(B)* was detected in 11 MRSA beside 8 isolates MSSA. The *erm(B)* was detected in 19 isolates (76%) (11 isolates MRSA and 8 isolates MSSA). *erm(C)* was detected in 15 MRSA isolates and in 10 of MSSA isolates. Both *erm(A) and erm(B)* were detected in 10 MRSA and 9 MSSA. Also, both *erm(B) and erm(C)* were detected in 11 MRSA and 8 MSSA. All three genes e*rm(A), erm(B) and erm(C)* were detected in 10 MRSA and 9 MSSA. A clear improved synergistic impact was observed when vitamin C and vitamin K_3_ were tested in combination with either erythromycin or azithromycin antibiotics for therapy.

According to clindamycin resistance phenotype, our results indicated three different phenotypes, Inducible MLSB, Constitutive MlSB and MS or NEG that includes 31, 39 and 7 isolates respectively, that were represented as follows: (25) MRSA, (6) MSSA, (30) MRSA, (9) MSSA and (0) MRSA and (7) MSSA, respectively.

The results of another study conducted in Texas, where the cMLS phenotype was the predominant resistance phenotype (41.7%) and both iMLS and MS phenotypes were just 3.3% each, are somewhat consistent with the findings of our study. Numerous investigations revealed that among MRSA, constitutive phenotypic resistance was more common than inducible resistance [20, 41, 42]. Additionally, our findings were consistent with those of [43], who discovered that 28.94% of erythromycin resistant isolates exhibited inducible resistance, whereas 34.12% exhibited constitutive resistance. According to [39], 30% of *S. aureus* was clindamycin resistant, with constitutive resistance occurring more frequently than inducible resistance (17.14% and 13.71%, respectively). In Europe, where the inducible resistance phenotype was predominated in MSSA, there was a high frequency of constitutive resistance in MRSA isolates (93%) [41].

According to Bottega et al. (2014), [44], MRSA had a higher prevalence of constitutive and inducible resistance than MSSA (68.9 vs 4.5%, 10.3% vs 7.2%). However, the findings of our investigation are quite dissimilar from those of a study conducted by Zachariah and colleagues, in which the MS phenotype was shown to be the most common resistance phenotype, followed by the iMLS phenotype and the cMLS phenotype (Zachariah et al., 2016) [45]. Another study conducted in Serbia found that the majority of the collected isolates (33.4%) had the iMLS phenotype, followed by the cMLS phenotype (8.9%) [46]. They attributed the increased usage of macrolides and lincosamides in healthcare settings to the high incidence of the iMLS phenotype [47]. According to diverse parameters including geographic location and demographic changes, the MLS resistance phenotype, whether constitutive or inducible, may differ greatly [47].

An antibiogram analysis of erythromycin, azithromycin, spiramycin, and clindamycin was conducted on 150 Staphylococcus sp. isolates obtained from diverse clinical specimen in a study with a procedure identical to ours. 54 (36%) of the 150 *Staphylococcus* sp. isolates that were collected and examined were resistant to two or more of the tested macrolides.

In 15 of the resistant isolates (27.8%), the inducible macrolide, lincosamide, and streptogramin type B resistance phenotype (iMLS) was found. Using polymerase chain reaction (PCR), the main genes encoding for macrolide resistance, such as erythromycin ribosomal methylase [*erm(A)* and *erm(C*)] and macrolide streptogramin resistance gene *msr(A*), were molecularly identified. It was discovered that 51.8%, 37.1%, and 11.1% of the resistant isolates, respectively, possessed one, two, and three types of resistance genes. However, the most often occurring gene was *erm(C)* (81.5%), followed by *msr(A)* (42.6%) and *erm(A)* (35.2%). In conclusion, the study's genotypic analysis showed that most of the tested isolates had two or more macrolide resistance-coding genes, and 36% of them showed resistance to at least two of the most prevalent macrolide antibiotics used in the treatment of such serious pathogens, especially in patients with penicillin hypersensitivity, according to several international guidelines [46].

Additionally, among 100 Staphylococcal samples, MRSA (45%), MSSA (8%), MRCoNS (13%), and MSCoNS 34/100 (34%) were found, which is consistent with the findings of our study. Clindamycin resistance and Erm gene positivity were very statistically significantly correlated, and 100% of ERY-S and CL-S were *erm(B) & (B)* negative. The isolates' antibiotic susceptibility patterns were identified as follows: 53% of the isolates had clindamycin resistance. Resistance to erythromycin is 48% and to efoxitin is 57%. Resistance to ciprofloxacin was 62%, methicillin resistance was 57%, ampicillin resistance was 46%, cefamandole resistance was 83%, amoxyclav 40%, vancomycin resistance was 80%, and aztreonam resistance was 86%. Azithromycin and amoxycillin resistance were both 77%.

The MLSB resistance pattern (EryS ClinS) was found in 50 (50%) of the isolates, followed by the constitutive phenotype (EryR ClinR) of 29 (29%), and the inducible phenotype (EryR ClinInd) of 17 (17%), with the MSB phenotype (EryR ClinS) being the least common (4%). For the genes *erm(B)* and *erm(C),* only 63 strains were genotypically examined. 51 isolates (including 28 *S. aureus*) had the *erm(B)* gene.18 of their isolates were phenotypically constitutive MLSB, 10 were inducible MLSB, but the MSB phenotype was not found. Additionally, the *erm(B*) gene was found in 21 of their isolates, all of which were CNS.33 isolates, (15 S. aureus) of them had the *erm(C)* gene, of which 10 isolates had the phenotypically constitutive MLSB phenotype, seven isolates had inducible MLSB, and only three isolates had the MSB phenotype, five of the isolates had phenotypically constitutive MLSB, ten had inducible MLSB but no MSB phenotype, and 18 of the isolates had the *erm(C)* gene. Seven of them were inducible MLSB isolates, and 11 of them had constitutive MLSB phenotypes, but the MSB phenotype was not found [48].

Additionally, it was discovered that the MSB phenotype (EryR ClinS) was the least common, occurring in 4 (4% of cases), followed by constitutive MLSB resistance (EryR ClinR), 29 (29%), and inducible MLSB resistance (EryR ClinInd), 17 (17%). The findings from our study concurred with those from Coutinho et al. (2010) [49] who found that 46.7% of *Staphylococci* tested positive for cMLSB, 3.3% for iMLSB, and 3.3% for MSB. The outcome, however, contradicts Pal et al. (2010) [50] They disagree with Deotale et al. (2010) [51] because their study revealed that 36 (14.5%) isolates exhibited inducible clindamycin resistance, 9 (3.6%) gave constitutive resistance, and other strains 35 (14.1%) displayed MS phenotype. Their study also revealed that constitutive resistance was demonstrated in (46.97%), inducible clindamycin resistance in (23.48%), and MS (29.53%) [48].

Contrary to our findings, a prior investigation (Lyall et al., 2013) [52] found that all of the isolates were susceptible to the antibiotic vancomycin, and the results of the El Mongy study of antibiotic susceptibility patterns for the isolates supported this finding also.

Only 63 strains were genotypically tested for the genes *erm(B) and erm(C)* in [48] study, which is relevant to the genotyping analysis. 18 of the 51 isolates in which the *erm(B)* gene was first discovered were phenotypically distinct constitutive MLSB, 10 were inducible MLSB with no MSB phenotype. Additionally, the *erm (B)* gene was detected in 21 isolates that 10 were inducible MLSB, but MSB phenotype was not found. Additionally, the *erm(B)* gene was found in 21 isolates that were CNS; 10 of these isolates were phenotypically constitutive MLSB, 7 were inducible MLSB, and only 3 were MSB phenotype. The *erm(C)* gene was found in 33 isolates (15 S. aureus), and it was found in 33 isolates overall. Additionally, the *erm(C)* gene was found in 18 isolates, of which CNS 11 isolates were phenotypically constitutive *MLSB* and 7 isolates were inducible *MLSB*, but MSB phenotype was not observed. Of these, 5 isolates were phenotypically constitutive *MLSB*, 10 isolates were inducible MLSB, but MSB phenotype was not detected [48].

In a different investigation, [53] discovered that *erm(C)* was more prevalent in the isolates with the constitutive phenotype and that *erm(B)* was only observed in (69%) of isolates. This finding agreed with that of Coutinho et al. (2010) [49] whose investigation found that 50.1% of isolates contained one or more *erm* genes. *erm(C)* and *erm(B)* were found in 29 and 3 isolates, respectively. Additionally, a different study's findings contradict ours because six *Staphylococcus aureus* strains isolated from cystic fibrosis patients after treatment with azithromycin were also cross-resistant to erythromycin and azithromycin. All isolates possessed either the A2058G/U or A2059G alterations in the *rrl* genes, with the mutation present in the majority of the rRNA copies, but none of them had the *erm* or *msr(A)* genes. One strain had an extra mutation in the L22 ribosomal protein-encoding *rplV* gene [54].

MRSA detection was evaluated using the molecular technique and the disc diffusion agar assay. The E-test was used to evaluate the efficacy of linezolid, vancomycin, mupirocin, teicoplanin, fusidic acid, and rifampin. By performing a D-test followed by a PCR assay for the *erm(A), erm(B)*, and *erm(C)* genes coding for macrolide resistance, several phenotypes of macrolide-lincosamide-streptogramin B (MLSB) resistance were identified. Among imipenem, meropenem, and imipenem/oxacillin, the cefoxitin disc produced the best sensitivity value (100%) at the time. Linezolid and teicoplanin completely destroyed all isolates. Vancomycin, fusidic acid, and rifampin resistance was found in 6.2%, 1.5%, and 17.1% of the MRSA isolates, respectively. The majority of our study's findings included different resistance outlooks for 56 isolates, of which 20.6% showed two unique induction phenotypes (D and D +) and 45% displayed non-induction (HD, R) phenotypes. Both inducible and constitutive clindamycin-resistant isolates were shown to have higher prevalence of the *erm(A)* gene alone and in combination with *erm(C)* [55].

The six isolates had MICs above 128 ug/ml for both erythromycin and azithromycin. While the spiramycin MIC for strain UCN16 was lower (8 ug/ml), five strains were also resistant to this 16-member ring macrolide. Clindamycin, a streptogramin B, and quinupristin all had more prevalent MICs [54].

Since vitamin K_3_ is a lipophilic vitamin that dissolves in lipids, it can be used as an antibiotic. Our results clearly showed synergistic effect of Erythromycin in combination with vitamins K_3_ and C, Vitamin K_3_ was effective at MIC 64 and 128 µg/ml with tested isolates, half MIC of it with Erythromycin was responsible for inhibiting activity among isolates 57.14% (16 isolates), and 77.78% (21 isolates), respectively. At ¼ of concentration, efficiency changed slightly with 64 µg/ml; it was 60.71% but 128 µg/ml decreased by nearly 10% that recorded 66.67%. An enhanced antimicrobial effect considered when MICs of Azt in combination with Vit K_3_ were, at least, four-fold lower than MICs of Azt alone. The menadione (Vit k_3_) demonstrated a MIC = 64 μg/mL against different Staphylococcus aureus isolates. The interaction effect of Azithromycin in combination with VIT K_3_ resulted in a reduction of the MICs by ≥ fourfold in most of *S. aureus* tested. On the other hand, Vit C was effective at 500–10000, half of vit. C concentration combined with Erythromycin reported 60% bacterial growth inhibition ratio, and 80% growth inhibition at ¼ of vit. C concentration. At MIC 2000–4000, vit C at ½ of MIC gave 57.5% inhibition, followed by 65% at ¼ of MIC concentration. Two vitamins also were effective in combinations with Azithromycin, vitamin K_3_ give with ½ of 64 MIC resulted in 64.29%, 57.14%, 53.57% inhibition at ½, ¼, and of MIC, respectively. vitamin K_3_ at ½, ¼, and of 128 MIC give 48.14, 37.04, 55.56% inhibitory ratio respectively. vitamin C with ½, ¼, and of 500–1000 MIC resulted in 20, 50, 50%, respectively, these values increased with increasing MIC, it reached 65.7, 65.7, and 71.4% with ½, ¼, and of 2000–4000 MIC. Our results were confirmed y study that noted.

The various antibiotic resistance mechanisms include efflux pumps, which are ubiquitous proteins localized in the cytoplasmic membrane of all kind of cells. During the last two decades, numerous structurally diverse compounds have been studied and shown to have efflux-inhibitory activity. These include currently available drugs employed for other indications, as well as natural and synthetic molecules. Menadione (vitamin K_3_), is a fat-soluble vitamin that has long been recognized for its essential role in coagulation and, more recently, has been proposed as a key nutrient in the regulation of soft tissue calcification. Therefore, in a study aimed to evaluate the effect of menadione efflux pumps in multidrug resistant strains of *S. aureus. RN4220 harboring plasmid pUL5054 was used, which carries the gene encoding the msr(A) macrolide efflux protein; and IS-58, which possesses the TetK tetracycline efflux protein; 1199B resists hydrophilic fluoroquinolones *via* a NorA-mediated mechanism and wild strain 1199B. The possible inhibition of efflux pumps was evaluated by reduction of MIC of ethidium bromide (EtBr) and antibiotics due the possible inhibitory effect of these substances. Efforts have been directed for identification of EPIs from natural sources. Some of the detrimental effects on bacterial cells may be attributed to the detergent properties of menadione on account of their amphipathic structure that was observed in strains 4220 and IS58 indicating possible effect on efflux pump* [56].

Reported rates of QR-MRSA, MDR, and XDR strains were found in 59.4%, 73.9%, and 37.6% of isolates. The most frequent SCCmecs were SCCmecIV (36.5%) and SCCmecV (26.8%). 39 spa types were found, with the t021, t044, and t267 types being the most common in QR-MRSA isolates. The invasive, drug-resistant isolates and QR-MRSA were dominated by ST22 and ST30. The *norA, gyrA*, and *grlB* genes were significantly repressed in isolates incubated for 24 h, which was the most obvious change in gene expression brought about by vitamin K2. However, more than one gene was down-regulated by vitamin K2 at 24 h following treatment. Additionally, when compared to untreated isolates, a substantial decline in QR-MRSA-treated isolates was seen. In other words, QR-MRSA had less impact on the *norA, grlA, grlB*, *gyrA*, and *gyrB* genes, so, as in our results, vitamin K could be used as *Staphylococcus aureus* growth inhibitor [57].

Another study, similar to ours, used a gradual increase in menadione's subinhibitory concentration to test the antibiotic-modifying activity of the vitamin in multi resistant strains of *Staphylococcus aureus*, *Pseudomonas aeruginosa*, and *Escherichia coli*. In addition, the drug-moderating mechanisms of vitamin K_3_, cholesterol, and ergosterol were compared. The broth microdilution assay was used to measure the antibacterial impact and antibiotic-modifying activity. Menadione, cholesterol, and ergosterol all demonstrated modulatory activity at clinically meaningful concentrations, designating them as bacterial drug resistance modifiers because they decreased the MIC of the examined antibiotics [58].

In addition, [59]. Explored the antimicrobial effects of quercetin on *Staphylococcus aureus* and other bacteria at certain concentrations-at which it is soluble- and recorded the antioxidant vitamin C modifies these activities. Their findings support the findings of our study about the usage of vitamin C. The bacteria under study that was the most sensitive was *S. aureus*. 90 M quercetin reduced S. aureus growth to 75% of the value for a control culture after 12 h of culture. The growth of *S. aureus* was dramatically reduced to 3% of that of the control culture treated with vitamin C alone when 1 mM vitamin C was coupled with 90 M quercetin. *S. aureus* growth was also suppressed by vitamin C alone, and at 5 mM, it was totally blocked. Vitamin C's stabilizing impact on quercetin helps to explain why it increases the antibacterial activity of quercetin. Even if vitamin C's acidity helps to stop *S. aureus* from growing, neutralized vitamin C effectively stops the growth even in the absence of quercetin. The findings imply that vitamin C has an impact on *S. aureus* metabolism and that these modifications are likely to be responsible for the observed growth suppression. Although vitamin C itself is a potent antioxidant, its aerobic metabolism makes bacterial cells are more susceptible to oxidative stress [59].

Additionally, a study that employed the agar well diffusion method to test the antibacterial activity of vitamin C on both Gram-positive and Gram-negative bacteria at various dosages (5–20 mg/ml), temperatures (4°C, 37°C, and 50°C), and pH levels (3, 8, and 11). Vitamin C concentration was necessary to limit the growth of all bacterial strains. Gram-positive bacteria, such as *Bacillus licheniformis*, *Staphylococcus aureus*, and *Bacillus subtilis,* as well as Gram-negative bacteria, such as *Proteus mirabilis, Klebsiella pneumoniae, Pseudomonas aeruginosa*, and *Escherichia coli* were all significantly inhibited by vitamin C. At a variety of pH levels and temperatures, vitamin C stability was observed at an acidic pH, all bacterial strains were significantly resistant to the antibacterial effects of vitamin C. The stability of vitamin C was unaffected by temperature changes. The results showed that vitamin C is a powerful and secure antibacterial agent [60].

In a different investigation, the Kirby-Bauer disc diffusion assay was used to assess the synergistic effects of antibiotics and stock solutions of vitamins. Water-soluble and fat-soluble vitamins, respectively, were dissolved in distilled water and propylene glycol, respectively. The water-soluble vitamins B1 (thiamine), B2 (riboflavin), B6 (pyridoxine), B12 (methyl cobalamin), and C (ascorbic acid) were used in final concentrations of 10 mg/mL, and the fat-soluble vitamins A (retinol), D (cholecalciferol), E (tocopherol), and K (menadione) were used in final concentrations of 0.1 mg/mL, respectively [61].

The study's findings showed that, whereas vitamins B1, B2, and B12 displayed impressive synergistic efficacy with linezolid against MRSA, vitamins K_3_ and E had good synergistic activity with piperacillin/tazobactam, imipenem, and doripenem against A. baumannii. Further research revealed that vitamin B1 worked better against MRSA when combined with oxacillin, tetracycline, rifampicin, and linezolid. While the water-soluble vitamins B1, B2, and B12 were effective against MRSA, but not A. baumannii, the fat-soluble vitamins E and K_3_ showed good synergism against Gram-negative A. baumannii. This synergistic action of vitamins with antibiotics may be used as a tool to treat MDR superbugs, with further evaluation required at a molecular level [61].

In another study, *Pseudomonas aeruginosa*, was tested by disc diffusion method using (12) different antibiotics. The results showed a different percentage of resistance to each antibiotic as (Gentamycin, amikacin, ampicillin, bacitracin, Ciprofloxacin, Norfloxacin, chloramphenicol, erythromycin, tetracycline, streptomycin, tobramycin, Trimethoprim sulfamethoxazole). The results revealed that Ciprofloxacin was the most effective antibiotic against bacterial isolates followed by amikacin and then by Norfloxacin, and the isolates are completely resistant to both erythromycin and tetracycline. Twelve isolates were selected to detect the effect of ascorbic acid when combined with antibiotics and tested by using disk diffusion assay. Various concentrations of the ascorbic acid were used, starting from (1 to 22.2 mg). The results showed that there is a synergistic interaction between vitamin C and most of the antibiotics, Also, the synergistic effect increases with increasing the concentration of the vitamin. The antibiotic chloramphenicol had the greatest effect, as the area of inhibition increased in 11 out of 12 isolates. Also, the tests showed that ascorbic acid had an antagonistic effect on some antibiotics, such as norfloxacin and tobramycin, where the inhibition area decreased in 9 and 8 isolates, respectively [62]. In contrast to our results, a study aimed to investigate the effects of ascorbic acid on antibiotic susceptibility of major bovine mastitis pathogens, including *Staphylococcus aureus, Streptococcus dysgalactiae, Streptococcus uberis, Streptococcus agalactiae*, and *Escherichia coli*, Minimum Inhibitory Concentrations (MICs) were determined by E-test method. The presence of 10 mM ascorbic acid decreased the MICs of penicillin and ampicillin, but it increased the MICs of erythromycin, kanamycin, streptomycin, and ciprofloxacin for all tested strains. Besides, ascorbic acid specifically reduced the MICs of tetracycline for gram-positive bacteria and chloramphenicol for Gram negative bacteria [63].

## Conclusion and recommendation

Multi Drug Resistant *Staphylococcus aureus* bacteria had different resistance mechanisms, *erm(A)*, *erm(B)* and *erm(C)* genes are considered an important mechanism of the resistance. The combination of antibiotics Erythromycin, Azithromycin and either vitamin K_3_ and C has a significant synergetic effect as antimicrobial agents for *Staphylococcus aureus* bacteria. We recommend the addition of vitamin C and K_3_ in the *Staphylococcus aureus* treatment protocol regimen.

### Supplementary Information

Below is the link to the electronic supplementary material.

**Additional file 1.**

## Data Availability

Any raw data files be needed in another format are available from the corresponding author upon reasonable request. Source data are provided with this paper.
