# Supplementary material for: Genetic diversity of macrolides resistant Staphylococcus aureus clinical isolates and the potential synergistic effect of vitamins, C and K3
Source: BMC Microbiol. 2024 Jan 20;24:30. doi: 10.1186/s12866-023-03169-1 (PMC10799532; doi:10.1186/s12866-023-03169-1)
Supplement: Supplementary file 1 — Additional file 1. [file 12866_2023_3169_MOESM1_ESM.zip › Figure after merge 24-11-2023.docx]

**Figure (5):**

**(A)**


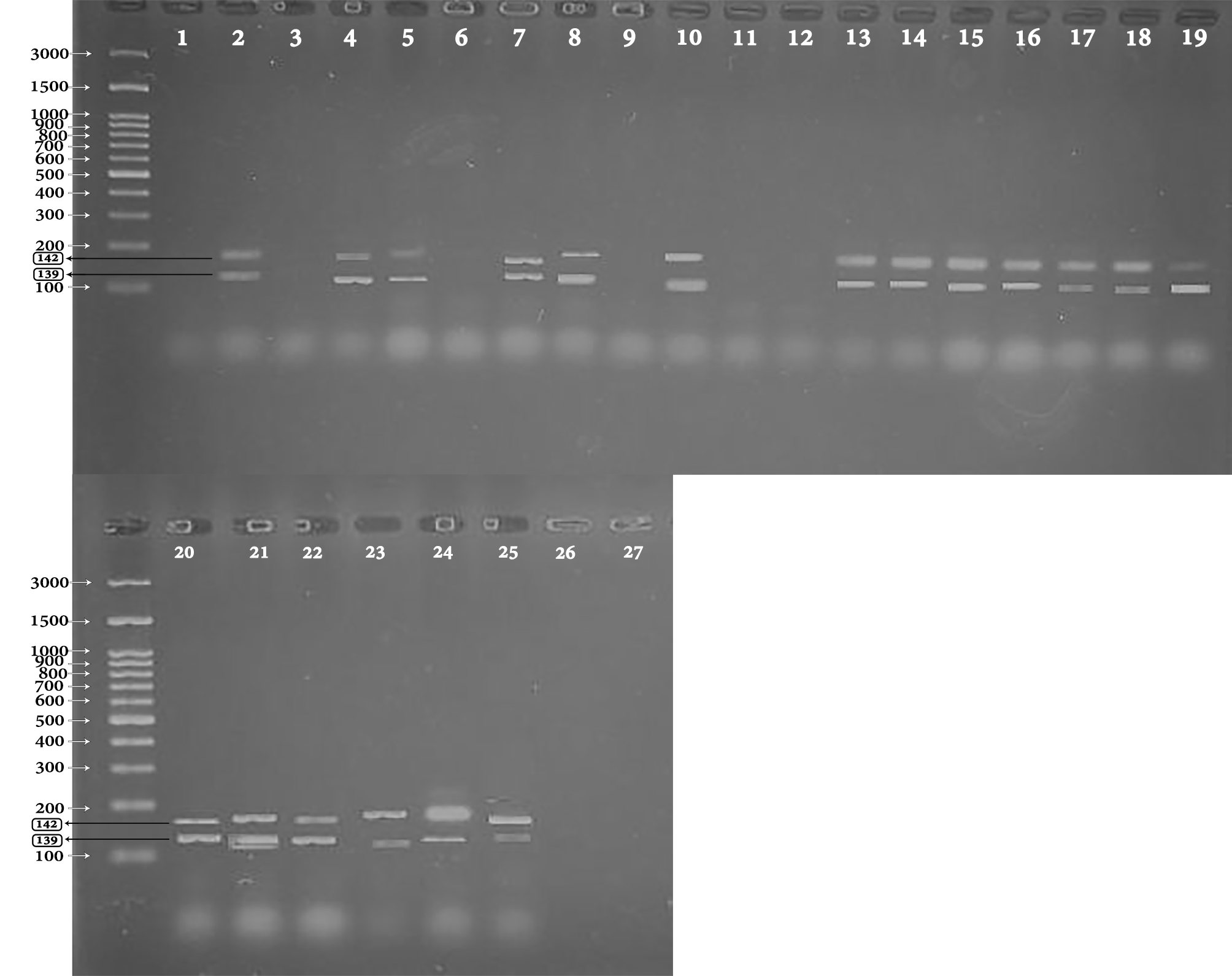


A: Agarose Gel electrophoresis of (*erm A* & *erm B*) genes at (139, 142bp).

ladder lane (m) is 1 Kb,

bacterial samples lanes from (1 to 19) are coding for isolates A, 1A, 19, 300, 308, 89^+^, 80, 36, 325, 305, 301, 44A, 3A, & 12A ,lanes from (20 to 25) are coding for isolates 28A, 317, 82A, 24, 4A, & 41A respectively.

**(B)**


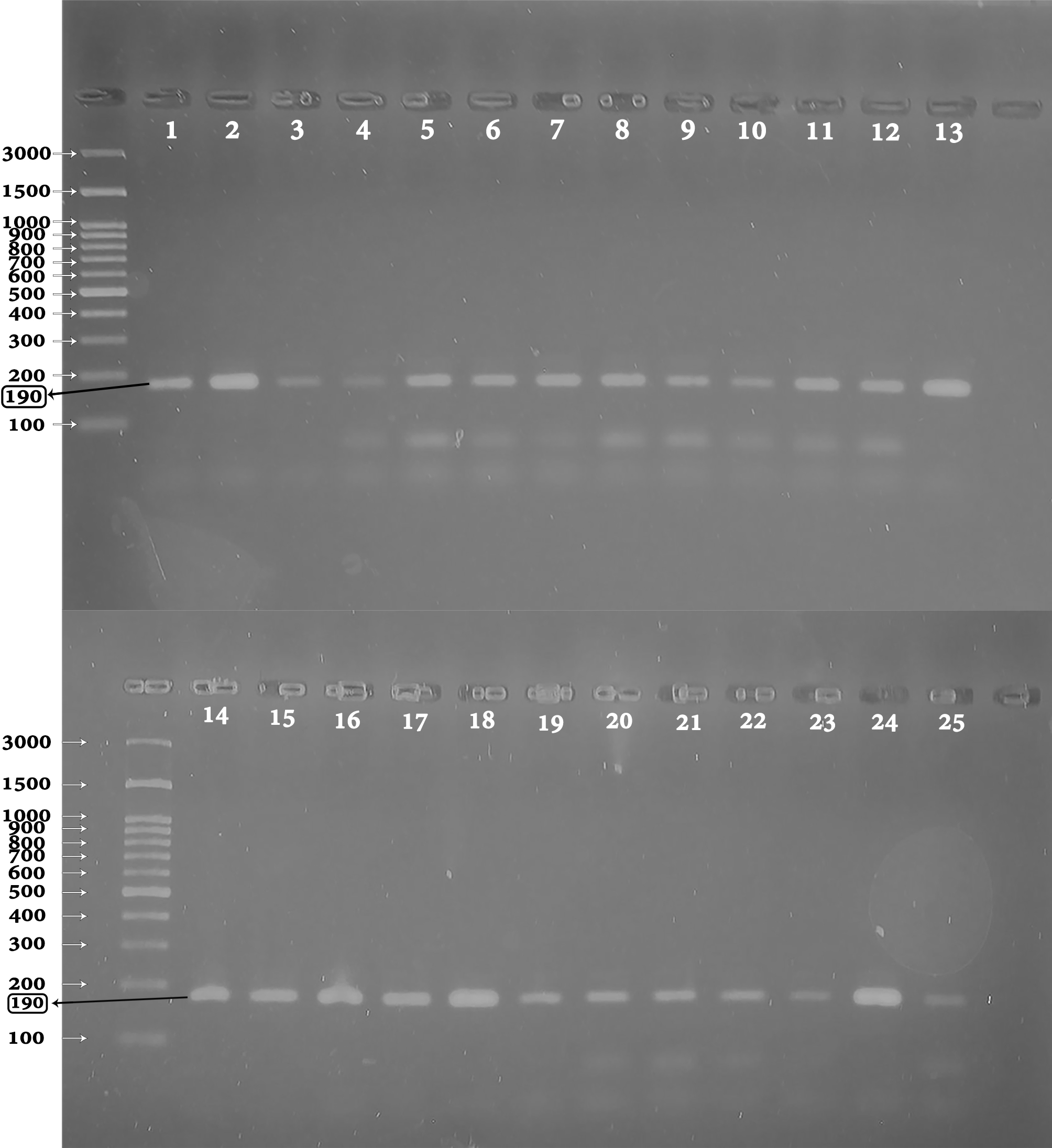


B: Agarose Gel electrophoresis of *erm(c)* gene at (190bp).

ladder lane (m) is 1 Kb.

Bacterial samples lanes from (1 to 13) are coding for isolates A, 1A, 19, 300, 308, 89^+^, 80, 36, 325, 305, 301, 44A & 32A, lanes from (14 to 25) are coding for isolates 38A, 45A, 46A, 43A, 3A, 12A, 28A, 317, 82A, 24, 4A & 41A respectively.

**(C)**

**
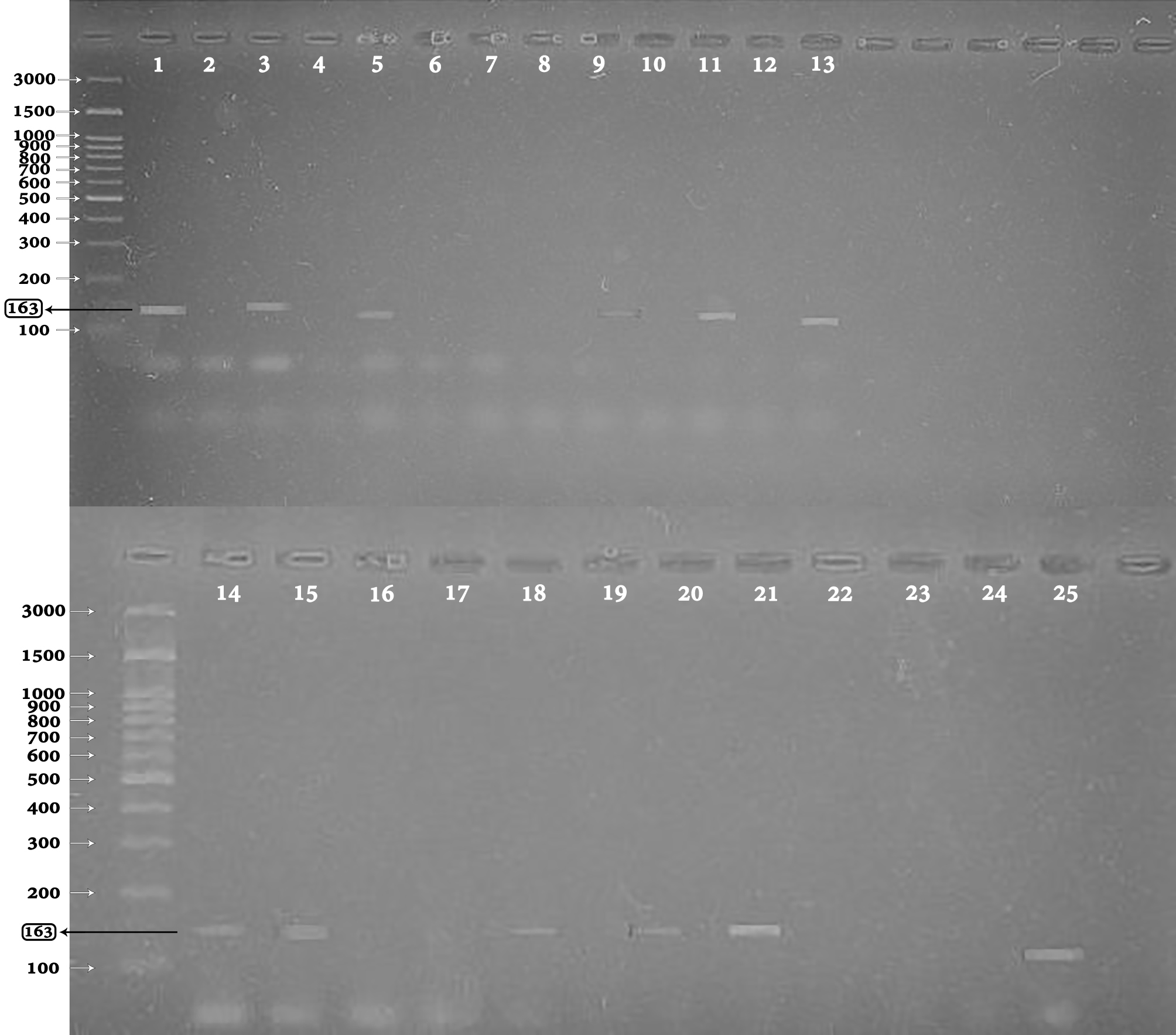
**

**C**: Agarose gel electrophoresis of *msr(A*) gene at (163bp).

Ladder lane (M) is (1 Kb).

Bacterial samples lanes from (1 to 13) are coding for isolates A, 1A, 19, 300, 308, 89^+^, 80, 36, 325, 305, 301, 44A & 32A, lanes from (14 to 25) are coding for isolates 38A, 45A, 46A, 43A, 3A, 12A, 28A, 317, 82A, 24, 4A & 41A respectively.

**(D)**


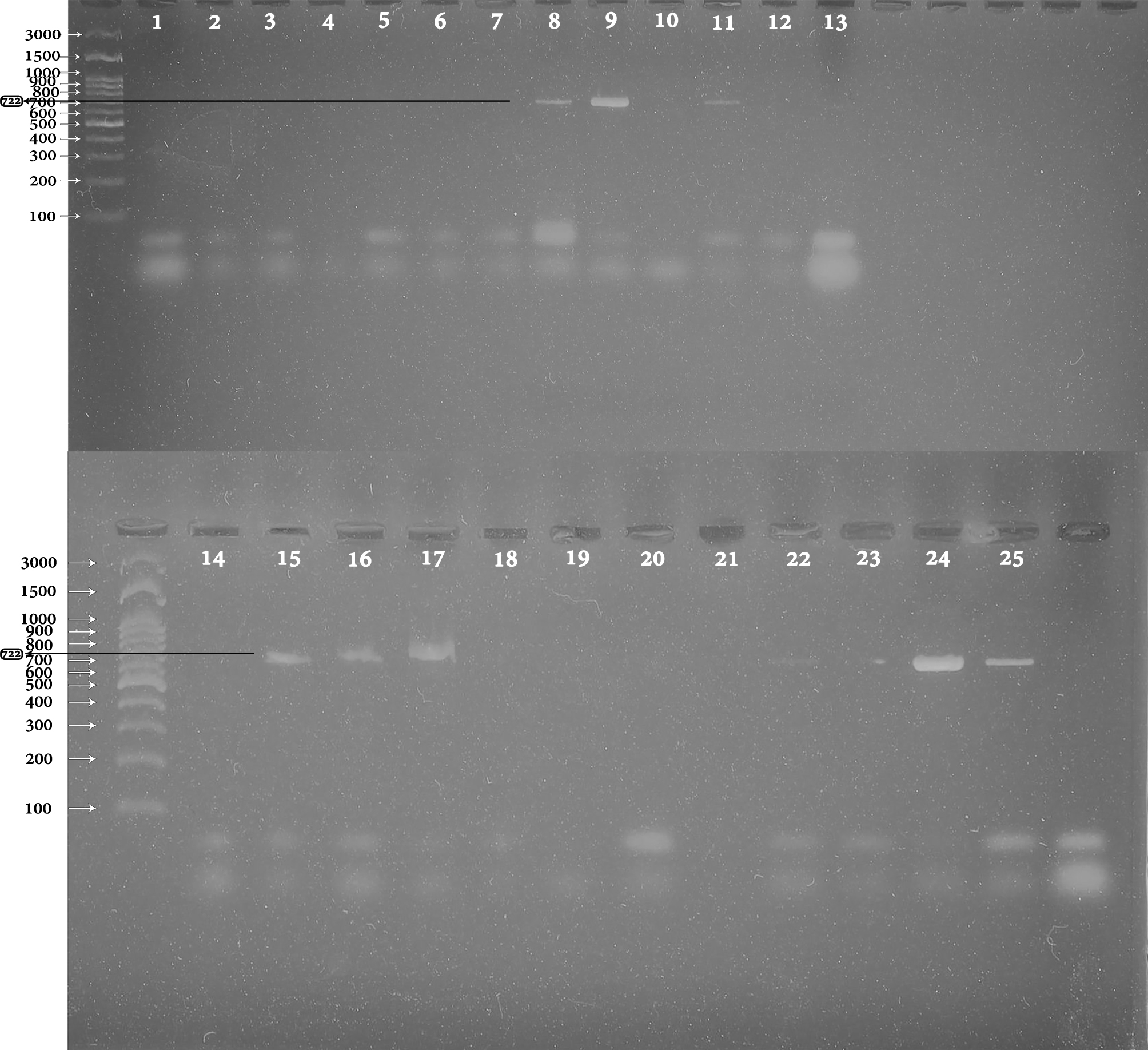


**D**: Agarose gel of electrophoresis of *mph(c)* gene at (722bp).

ladder lane (M) is (1 Kb).

Bacterial Samples lanes from (1 to 13) are coding for isolates A, 1A, 19, 300, 308, 89^+^, 80, 36, 325, 305, 301, 44A & 32A, lanes from (14 to 25) are coding for isolates 38A, 45A, 46A, 43A, 3A, 12A, 28A, 317, 82A, 24, 4A & 41A respectively.
