# Supplementary material for: Genetic diversity of macrolides resistant Staphylococcus aureus clinical isolates and the potential synergistic effect of vitamins, C and K3
Source: BMC Microbiol. 2024 Jan 20;24:30. doi: 10.1186/s12866-023-03169-1 (PMC10799532; doi:10.1186/s12866-023-03169-1)
Supplement: Supplementary file 1 — Additional file 1. [file 12866_2023_3169_MOESM1_ESM.zip › Figure before merge 24-11-2023.docx]

**(Supplementary file)**

**The full uncropped Gels and Blots images**

**Figure (5):**


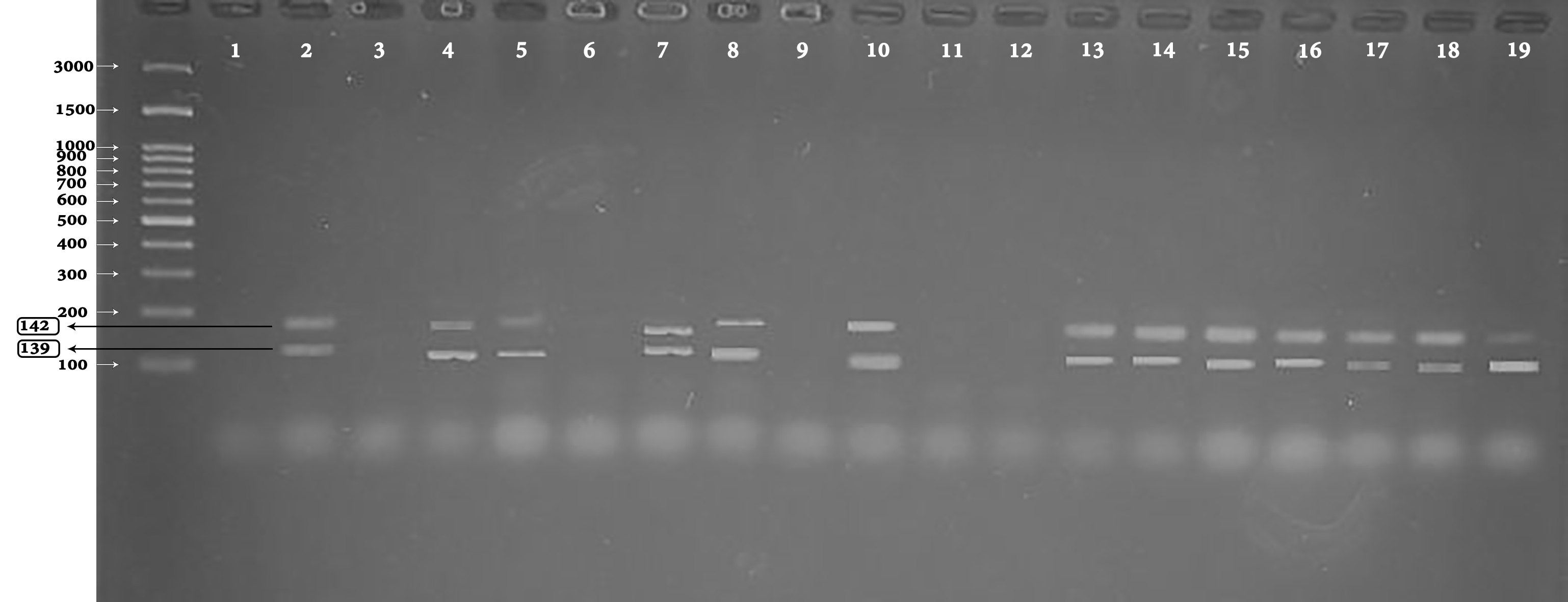


**Fig (5-A)**: Agarose Gel electrophoresis of (*erm A* & *erm B*) genes at (139, 142bp).

ladder lane (m) is 1 Kb,

bacterial samples lanes from (1 to 19) are coding for isolates A, 1A, 19, 300, 308, 89^+^, 80, 36, 325, 305, 301, 44A, 3A, & 12A, respectively.


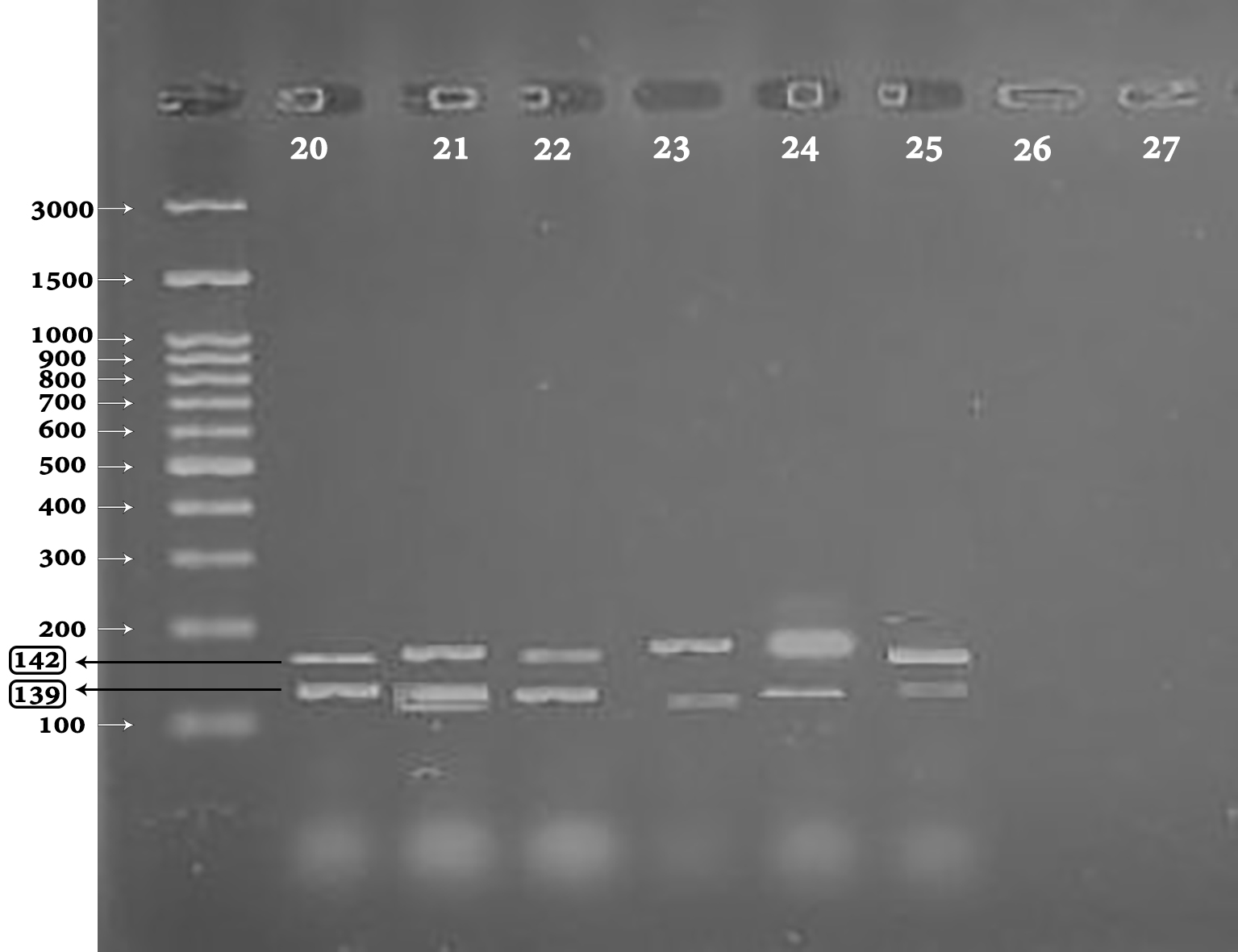


**Fig (5-A):** Agarose Gel electrophoresis of (*erm A* & *erm B*) genes at (139, 142bp).

ladder lane (m) is 1 Kb,

bacterial samples lanes from (20 to 25) are coding for isolates lanes from (20 to 25) are coding for isolates 28A, 317, 82A, 24, 4A, & 41A respectively


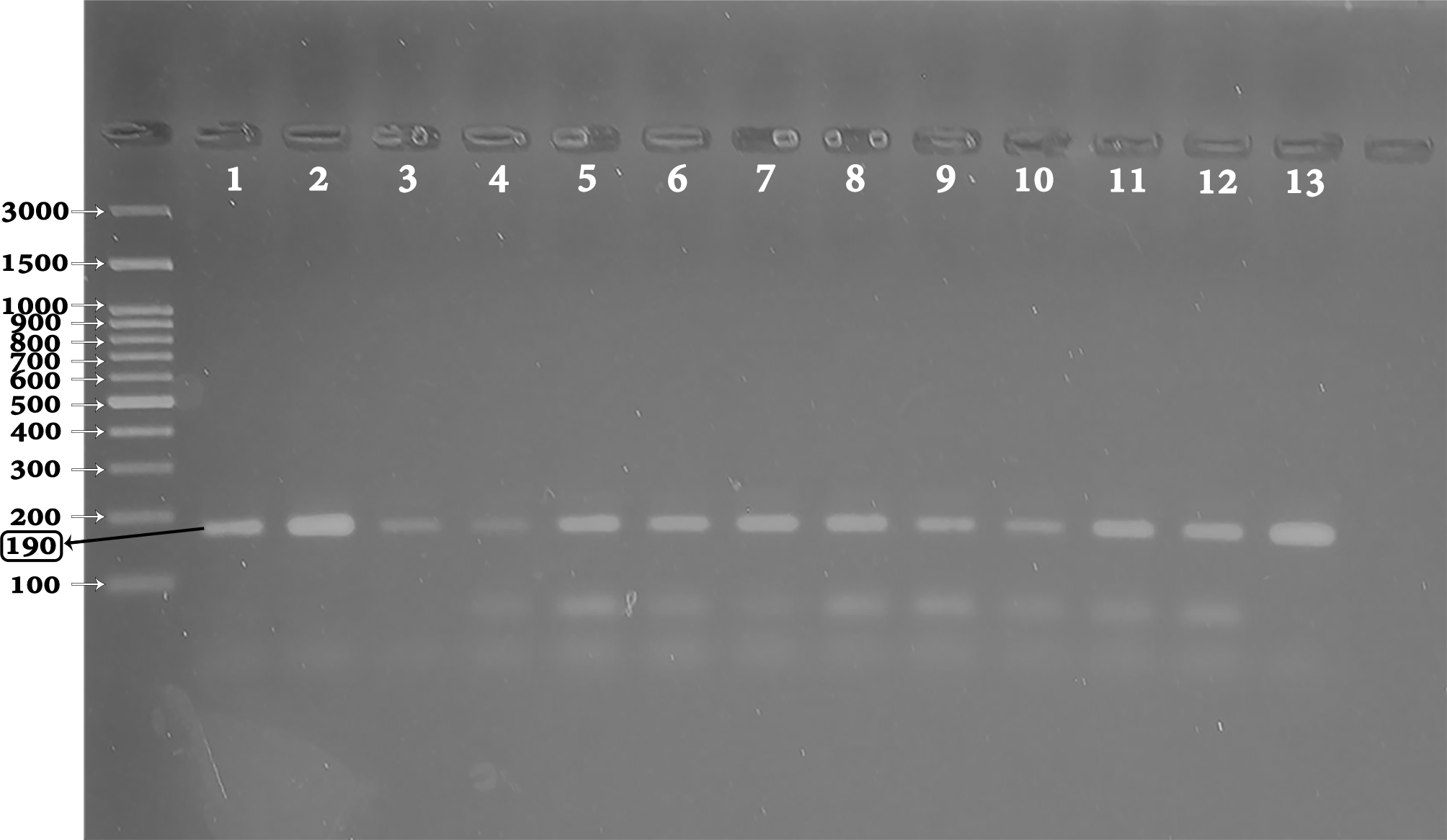


**Figure(5-B):** Agarose Gel electrophoresis of *erm(c)* gene at (190bp).

ladder lane (m) is 1 Kb.

Bacterial samples lanes from (1 to 13) are coding for isolates A, 1A, 19, 300, 308, 89^+^, 80, 36, 325, 305, 301, 44A & 32A, respectively


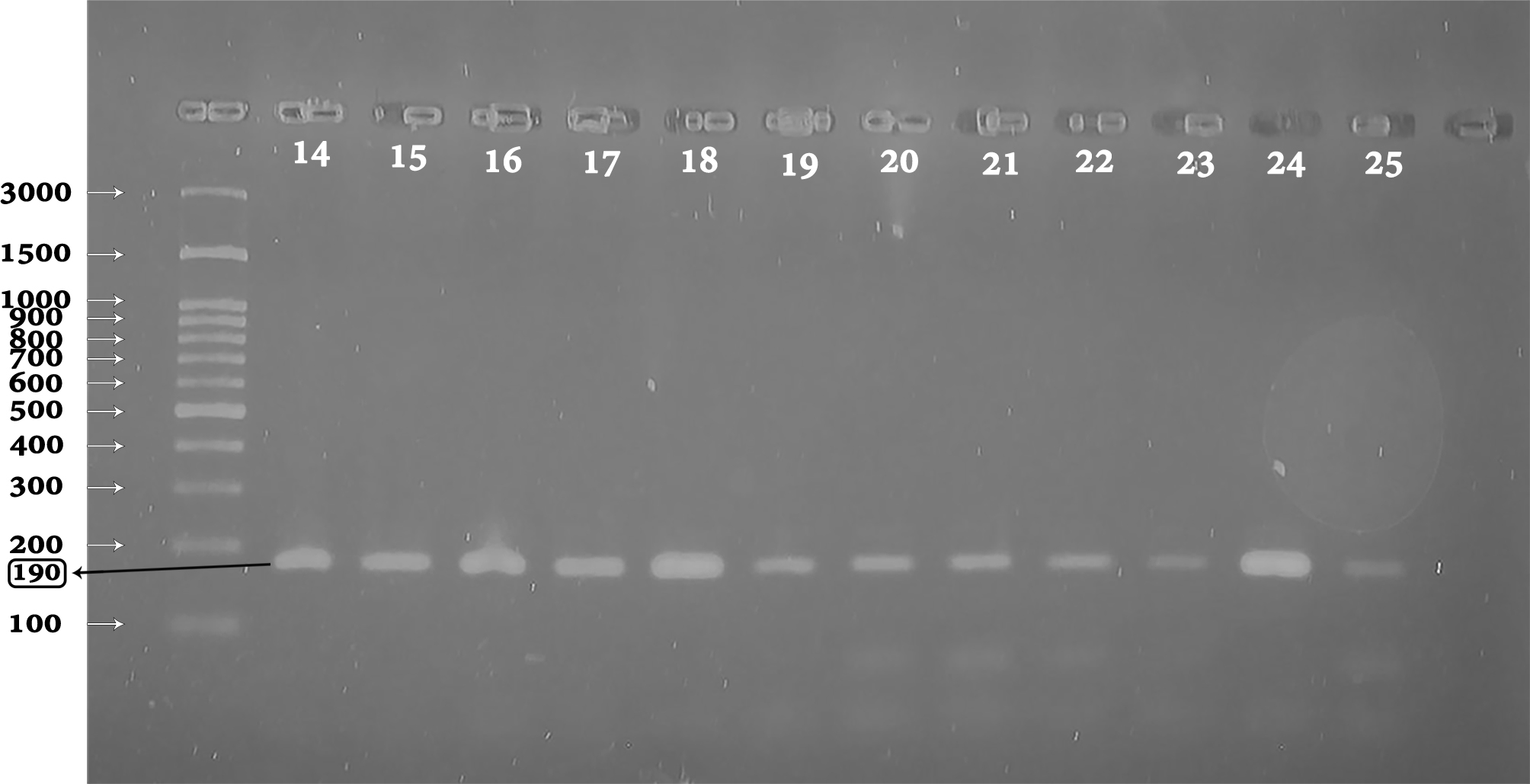


**Figure(5-B):** Agarose Gel electrophoresis of *erm(c)* gene at (190bp).

ladder lane (m) is 1 Kb.

Bacterial samples lanes from (14 to 25) are coding for isolates 38A, 45A, 46A, 43A, 3A, 12A, 28A, 317, 82A, 24, 4A


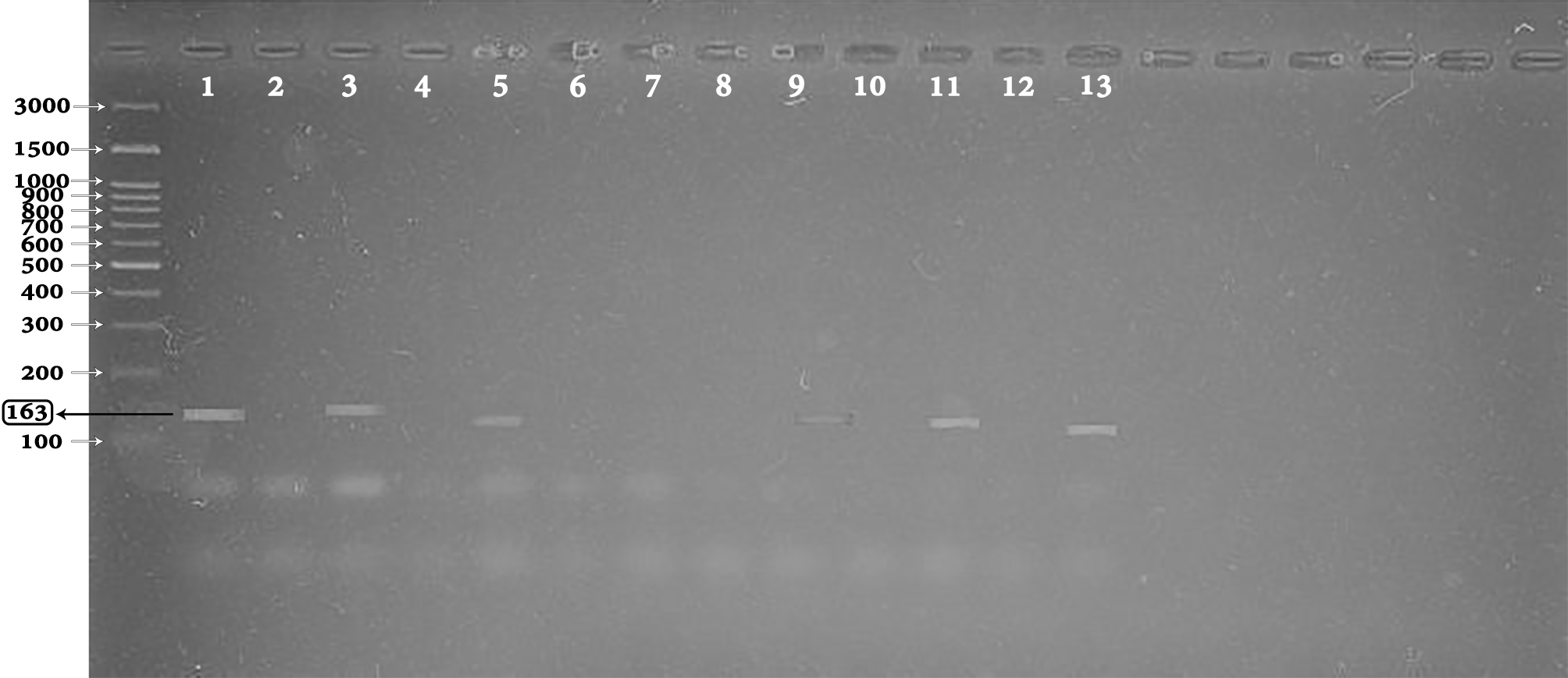


**Figure(5-C)**: Agarose gel electrophoresis of *msr(A*) gene at (163bp).

Ladder lane (M) is (1 Kb).

Bacterial samples lanes from (1 to 13) are coding for isolates A, 1A, 19, 300, 308, 89^+^, 80, 36, 325, 305, 301, 44A & 32A, respectively


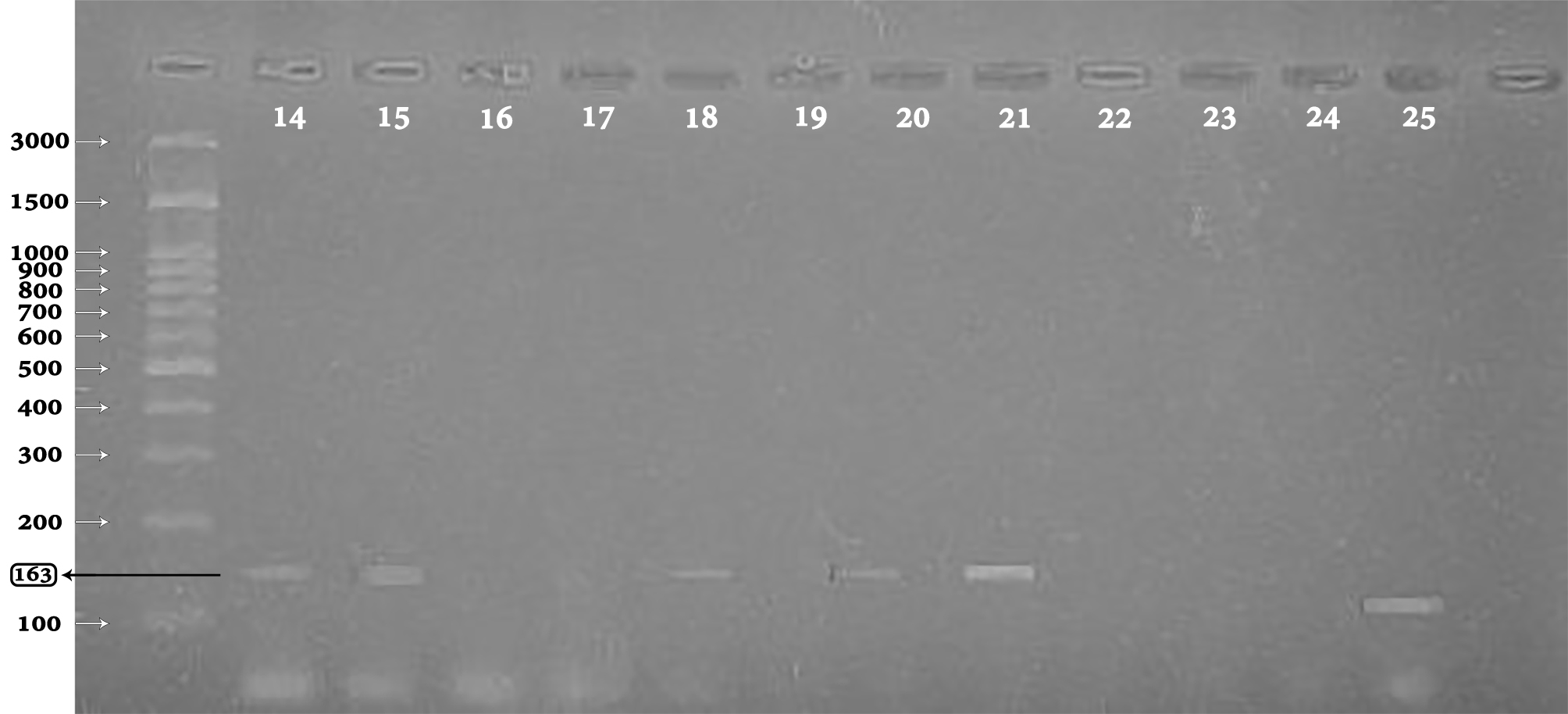


**Figure (5- C)**: Agarose gel electrophoresis of *msr(A*) gene at (163bp).

Ladder lane (M) is (1 Kb).

Bacterial samples lanes from (14 to 25) are coding for isolates 38A, 45A, 46A, 43A, 3A, 12A, 28A, 317, 82A, 24, 4A & 41A respectively


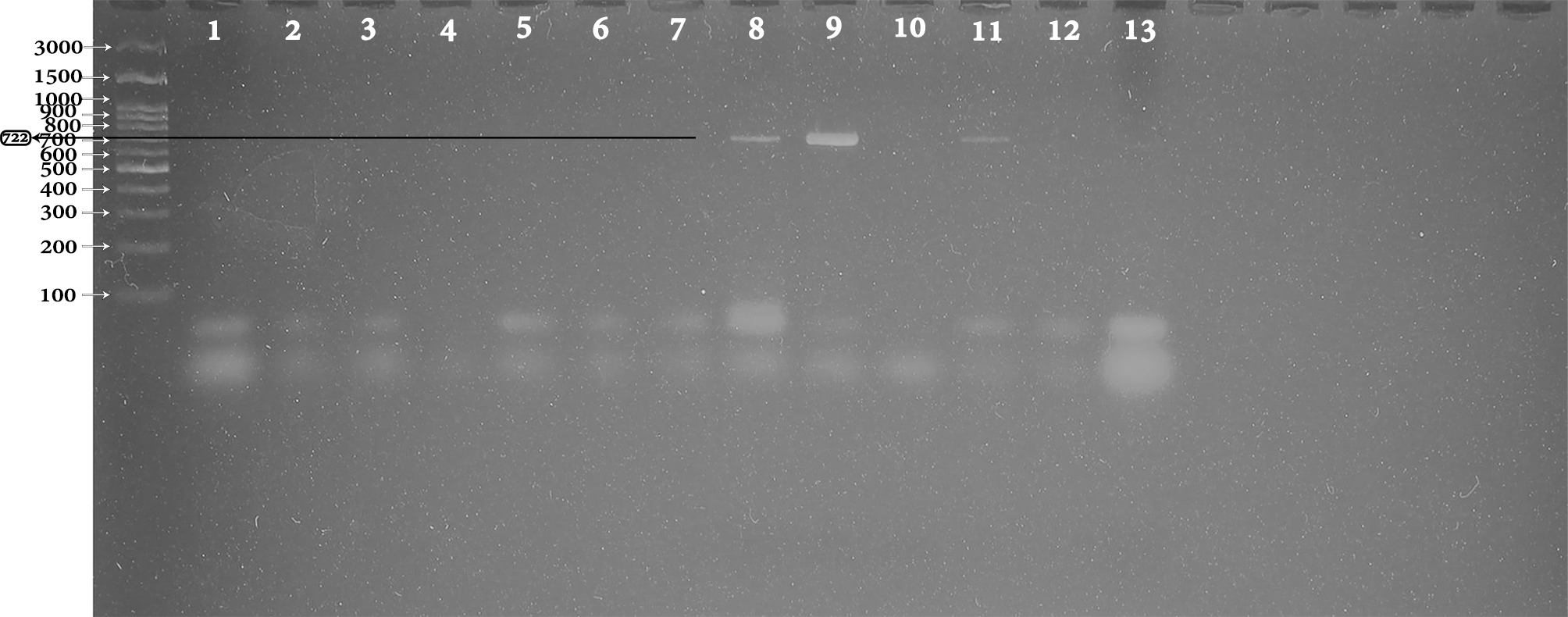


**Figure(5-D)**: Agarose gel of electrophoresis of *mph(c)* gene at (722bp).

ladder lane (M) is (1 Kb).

Bacterial Samples lanes from (1 to 13) are coding for isolates A, 1A, 19, 300, 308, 89^+^, 80, 36, 325, 305, 301, 44A & 32A, respectively


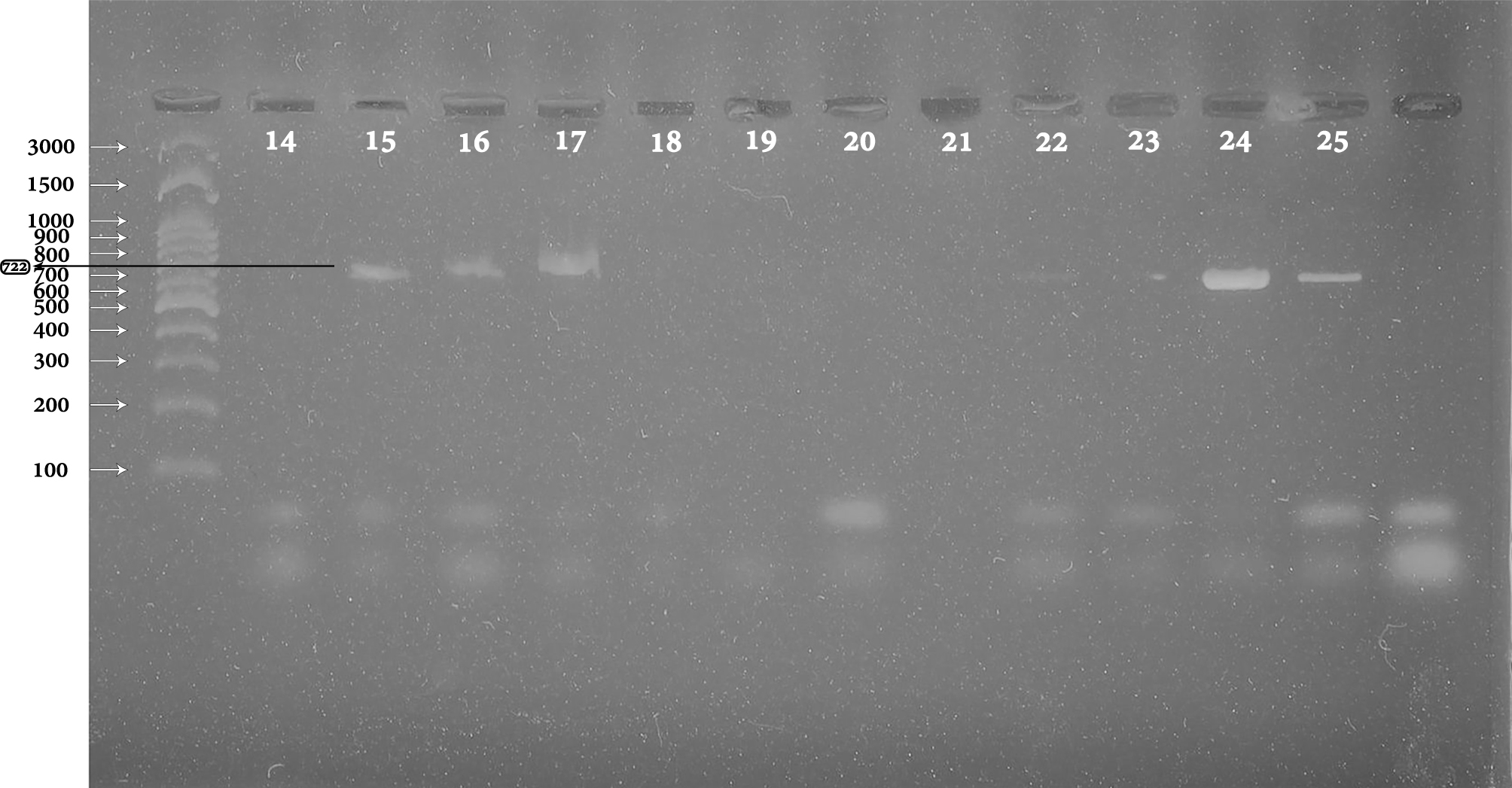


**Figure(5-D)**: Agarose gel of electrophoresis of *mph(c)* gene at (722bp).

ladder lane (M) is (1 Kb).

Bacterial Samples lanes from (14 to 25) are coding for isolates 38A, 45A, 46A, 43A, 3A, 12A, 28A, 317, 82A, 24, 4A & 41A respectively
